# A novel STING variant triggers endothelial toxicity and SAVI disease

**DOI:** 10.1084/jem.20232167

**Published:** 2024-07-02

**Authors:** Erika Valeri, Sara Breggion, Federica Barzaghi, Monah Abou Alezz, Giovanni Crivicich, Isabel Pagani, Federico Forneris, Claudia Sartirana, Matteo Costantini, Stefania Costi, Achille Marino, Eleonora Chiarotto, Davide Colavito, Rolando Cimaz, Ivan Merelli, Elisa Vicenzi, Alessandro Aiuti, Anna Kajaste-Rudnitski

**Affiliations:** 1 San Raffaele Telethon Institute for Gene Therapy, Istituto di Ricovero e Cura a Carattere Scientifico (IRCCS) San Raffaele Scientific Institute, Milan, Italy; 2 Vita-Salute San Raffaele University, IRCCS San Raffaele Scientific Institute, Milan, Italy; 3 Pediatric Immunohematology and Bone Marrow Transplantation Unit, IRCCS San Raffaele Scientific Institute, Milan, Italy; 4 Viral Pathogenesis and Biosafety Unit, IRCCS San Raffaele Scientific Institute, Milan, Italy; 5 Department of Biology and Biotechnology, University of Pavia, Pavia, Italy; 6 Fondazione IRCCS Policlinico San Matteo, Pavia, Italy; 7 Unit of Pediatric Rheumatology, ASST Gaetano Pini-CTO, Milan, Italy; 8 R&I Genetics Srl, Padua, Italy

## Abstract

Gain-of-function mutations in STING cause STING-associated vasculopathy with onset in infancy (SAVI) characterized by early-onset systemic inflammation, skin vasculopathy, and interstitial lung disease. Here, we report and characterize a novel STING variant (F269S) identified in a SAVI patient. Single-cell transcriptomics of patient bone marrow revealed spontaneous activation of interferon (IFN) and inflammatory pathways across cell types and a striking prevalence of circulating naïve T cells was observed. Inducible STING F269S expression conferred enhanced signaling through ligand-independent translocation of the protein to the Golgi, protecting cells from viral infections but preventing their efficient immune priming. Additionally, endothelial cell activation was promoted and further exacerbated by cytokine secretion by SAVI immune cells, resulting in inflammation and endothelial damage. Our findings identify STING F269S mutation as a novel pathogenic variant causing SAVI, highlight the importance of the crosstalk between endothelial and immune cells in the context of lung disease, and contribute to a better understanding of how aberrant STING activation can cause pathology.

## Teaser

Valeri et al. identify a novel STING variant (F269S) that causes SAVI disease. They highlight the activation of interferon and inflammation across patient cell types and underscore the importance of the crosstalk between endothelial and immune cells in disease pathology.

## Graphical Abstract

**Figure:**
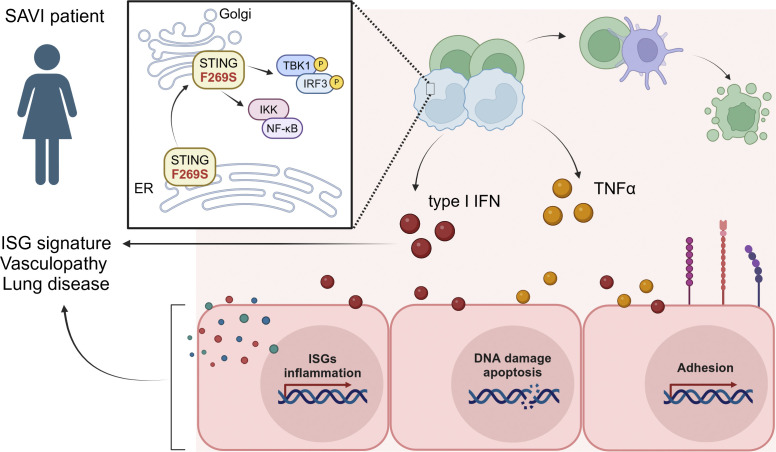

## Introduction

STING, also known as transmembrane protein 173 (TMEM173), is a cytosolic nucleic acid sensor that plays a central role in the innate immune response against invading pathogens by sensing microbial DNA and cyclic dinucleotides, as well as in the recognition of aberrant self-DNA (Ishikawa and Barber, 2008; Burdette et al., 2011; Yu and Liu, 2021; Abe et al., 2013). STING primarily localizes to the endoplasmic reticulum (ER) where it is activated by the 2′3′ cyclic GMP-AMP (cGAMP) produced by cGAMP synthase (cGAS) upon detection of cytosolic DNA (Yin et al., 2012; Ouyang et al., 2012; Wu et al., 2013; Ishikawa and Barber, 2008). Upon binding, STING undergoes conformational changes and translocates from the ER to the Golgi, where it orchestrates the activation of interferon (IFN) responses and proinflammatory gene induction (Yum et al., 2021; Abe and Barber, 2014; Shang et al., 2019; Zhong et al., 2008). The cGAS-STING pathway has been implicated in several monogenic autoimmune diseases, such as Aicardi–Goutières syndrome (AGS), where defects in AGS-causing genes lead to cytosolic nucleic acid accumulation and activation of cGAS-STING pathway (Yan, 2017; Pokatayev et al., 2016; Giordano et al., 2022). Furthermore, aberrant activation of the STING pathway due to its deficient retrograde movement from the Golgi to the ER has been associated with COPA syndrome (Steiner et al., 2022; Mukai et al., 2021; Lepelley et al., 2020; Deng et al., 2020). De novo or inherited gain-of-function (GOF) mutations in STING result in STING-associated vasculopathy with onset in infancy (SAVI), a rare autoinflammatory genetic disease characterized by constitutive STING trafficking and signaling independent of ligand binding (Liu et al., 2014; Jeremiah et al., 2014; Fremond et al., 2021). Over 80 SAVI patients have been identified so far with typical clinical features including neonatal-onset systemic inflammation, vasculopathic lesions affecting the lung and skin, interstitial lung disease (ILD), and respiratory failure. Increased phosphorylation of STAT1 and elevated expression of IFN-stimulated genes (ISGs) in patient blood cells have been observed (Jeremiah et al., 2014; Liu et al., 2014; Fremond et al., 2021), highlighting a constitutive activation of type I IFN response, which is characteristic of interferonopathies. Alterations in the immunological phenotype such as T cell cytopenia, reduced T cell proliferation, and reduction of the memory compartment are other common features of SAVI patients (Liu et al., 2014). SAVI mouse models have indicated that the negative effects of STING activation in T cells are IFN independent (Bouis et al., 2019; Cerboni et al., 2017; Warner et al., 2017; Liu et al., 2014). SAVI, together with COPA syndrome, is unique among type I interferonopathy in presenting with predominant lung involvement. However, the specific mechanisms through which STING promotes inflammation in the lung remain unclear. Different mouse models have been generated to gain mechanistic insight into lung pathology, revealing a central role of T cells in promoting lung inflammation (Warner et al., 2017; Luksch et al., 2019; Gao et al., 2022). Nevertheless, not all models fully recapitulate the IFN phenotype typical of the human disease. More recently, it has been demonstrated that endothelial cells expressing STING GOF are activated, suggesting that they may contribute to the initiation of inflammation (Gao et al., 2022; Gao et al., 2023, *Preprint*). However, the relative contribution of lung cells and cells of hematopoietic origin to the pulmonary phenotype is still under investigation.

Here, we present a de novo mutation located within the c-d-GMP binding domain of STING mapping proximate to interdimer contacts enabling STING multimerization, which leads to the development of SAVI disease. Through transcriptomic analysis we confirm upregulation of type I IFN responses and inflammation in multiple patient blood cells. Additionally, we demonstrate that the expression of STING F269S in both hematopoietic and non-hematopoietic cells triggers IFN responses independently of ligand binding, through translocation of the protein to the Golgi. Furthermore, STING F269S expression in T cell models leads to increased cell death, especially when exposed to TCR stimulation. Moreover, the expression of STING F269S in endothelial cells promotes their activation, and cytokines secreted by immune cells further enhance the activation and damage of endothelial cells. Lastly, we provide evidence of increased resistance of SAVI cells to viral infections. However, this resistance is associated with a lack of priming by exogenous stimulation, suggesting that excessive IFN signaling may trigger negative feedback regulation of the pathway.

## Results

### Clinical presentation

**Figure 1.**
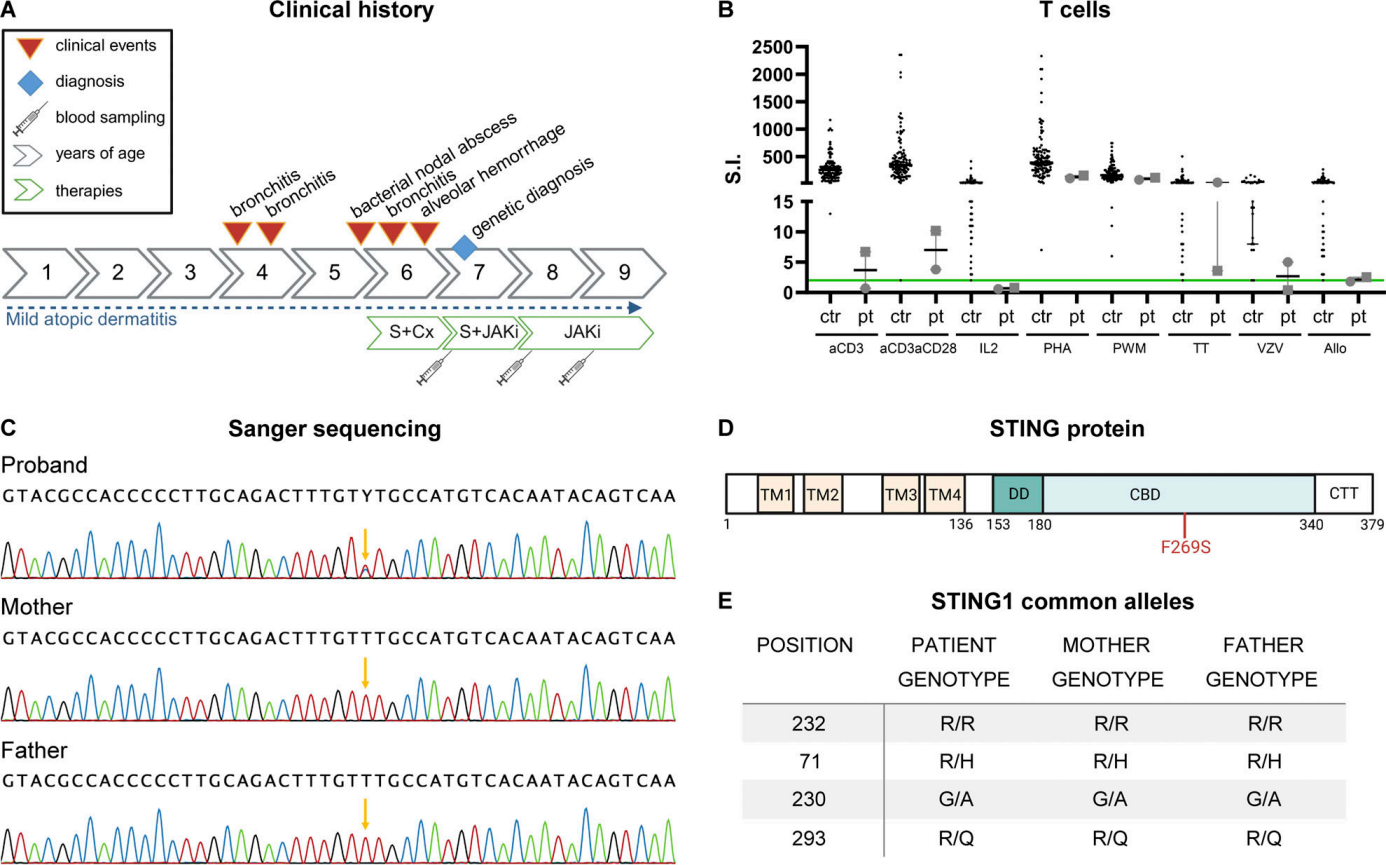
**Clinical presentation of the SAVI patient. (A)** Medical history timeline of the patient (S = steroids; Cx = cyclophosphamide; JAKi = JAK inhibitor). **(B)** Proliferative response to polyclonal mitogens (antiCD3, antiCD3/CD28, IL2) and antigens (TT: tetanus toxoid, VZV: varicella, Allo: alloantigens) of SAVI patient T cells. The response to each stimulus was compared between a control group of healthy individuals (ctr, black) and the patient (pt, gray). The patient has been evaluated before (gray dots) and after (gray squares) therapy. The green line indicates the cut-off for a positive response (S.I. = 2) (*n* = 30–115 healthy control samples; *n* = 2 SAVI patient samples). **(C)** Sanger sequencing highlights the de novo occurring mutation in STING1 gene in the SAVI patient and the WT sequence in her parents. **(D)** STING protein domains with highlighted the de novo mutation causing SAVI disease in the patient (TM = transmembrane domain; DD = dimerization domain; CBD = c-di-GMP binding domain; CTT = C-terminal tail; created with https://Biorender.com). **(E)** Genotype of STING1 common alleles in the SAVI patient and her parents.

The patient was born from non-consanguineous parents, at 41 gestational weeks, after an uneventful pregnancy. The neonatal period and neurological development were unremarkable. She received standard immunizations for age, without any complications. The patient has displayed atopic dermatitis since birth. During infancy, she experienced an abscess of the sub-mandibular lymph node treated with antibiotic therapy and three episodes of bronchitis. After the last one, when 5.5 years old, she manifested respiratory difficulties progressively worsening over 4 mo (Fig. 1 A). Eventually, she was admitted to the hospital due to important respiratory distress, needing oxygen support, and severe anemia (Hemoglobin [Hb] 5.6 g/dl). The CT scan showed multiple areas of increased density and ground glass opacities, ubiquitously distributed which, combined with anemia were suggestive of diffuse alveolar hemorrhage (DAH). The diagnosis was confirmed by broncho-alveolar lavage and was secondary to p-ANCA–associated vasculitis with >14,796 arbitrary units (A.U.) of anti-myeloperoxidase antibodies detected (normal value [n.v.] <20 A.U.). According to an immunological point of view, she showed: hypergammaglobulinaemia (IgG 19.9 g/l) at onset, with normal IgM and IgA, and absolute lymphopenia (1,543/mmc with n.v. 1,900–3,700), with a significant memory T cells deficiency more marked on CD8^+^CD45RO^+^ (16/mmc with n.v. 30–330) but involving also CD4^+^CD45RO^+^ T cells (105/mmc with n.v. 70–390) with naïve T cells showing a clear prevalence in the peripheral blood (Table 1). The lymphocyte proliferation test at onset showed a strong reduction of the TCR-mediated response to anti-CD3 and anti-CD3/anti-CD28 and IL-2 compared with healthy controls, while the response to polyclonal stimuli (PHA, pokeweed mitogen [PWM]) and to tetanus, varicella and alloantigens were present, although the antigen-specific response is poor, in line with the lack of memory T cells (particularly CD8^+^) (Fig. 1 B). The TCR-mediated response improved when tested 2 years after the onset when the state of hyperactivation was solved (Fig. 1 B). On the contrary, deficiency in the response to IL-2 stimulation persisted undetectable (Fig. 1 B). The patient was initially treated with high doses of steroids (IV methylprednisolone) and then shifted to cyclophosphamide, which induced remission. To establish if a genetic cause of the disease was present, a targeted panel of genes related to primary immunodeficiencies was analyzed, revealing the presence of a de novo missense variant in the *STING1* gene (c.806T>C; p.Phe269Ser) (Fig. 1, C and D), which led to the diagnosis of SAVI disease. After the genetic diagnosis of interferonopathy, the patient started baricitinib as maintenance therapy, which is still ongoing for>2 years, with good control of the underlying disease (Fig. 1 A). The human STING1 gene shows great heterogeneity in the human population, with R232, HAQ (R71H-G230A-R293Q), and H232 as the most common alleles (Patel and Jin, 2019; Froechlich et al., 2023). While R232 and HAQ have been reported as functional alleles, the H232 variant is severely impaired in STING function. The presence of these variants in the patient and her parents was additionally analyzed. Both present an R232/R232 WT genotype and are heterozygous carriers of the HAQ variant (Fig. 1 E), suggesting no impairment of canonical STING function.

**Table 1.** Peripheral blood lymphocyte subsets at onset and during follow up

Subset	Onset	+2 years	+3 years	Normal values^a^
Total leukocytes	4,900	6,600	6,300	4,400–9,500
Total lymphocytes	1,544	1,574	3,395	1,900–3,700
CD19^+^	452	267	615	270–860
CD3^+^	1,021	1,108	2,523	1,200–2,600
CD3^+^CD4^+^	638	728	1,652	650–1,500
CD3^+^CD8^+^	358	349	809	370–1,100
CD4/CD8 ratio	1.78	2.09	2.04	
CD16^+^CD56^+^	48	100	119	100–480
CD4^+^CD45RA^+^	543	648	1,305	1,200–3,700
CD4^+^CD45RO^+^	105	91	350	70–390
CD8^+^CD45RA^+^	367	389	850	450–1,500
CD8^+^CD45RO^+^	16	16	28	30–330
TCRα/β^+^ lymphocytes	671	759	1,839	
TCRγ/δ^+^ lymphocytes	12	20	29	
κ^+^ CD19^+^	241	144	339	
λ^+^ CD19^+^	206	120	270	
κ/λ ratio	1.2	1.2	1.3	

a Shearer et al., 2003.

### Single-cell transcriptomic reveals spontaneous upregulation of type I IFN and inflammation in patient blood cells

**Figure 2.**
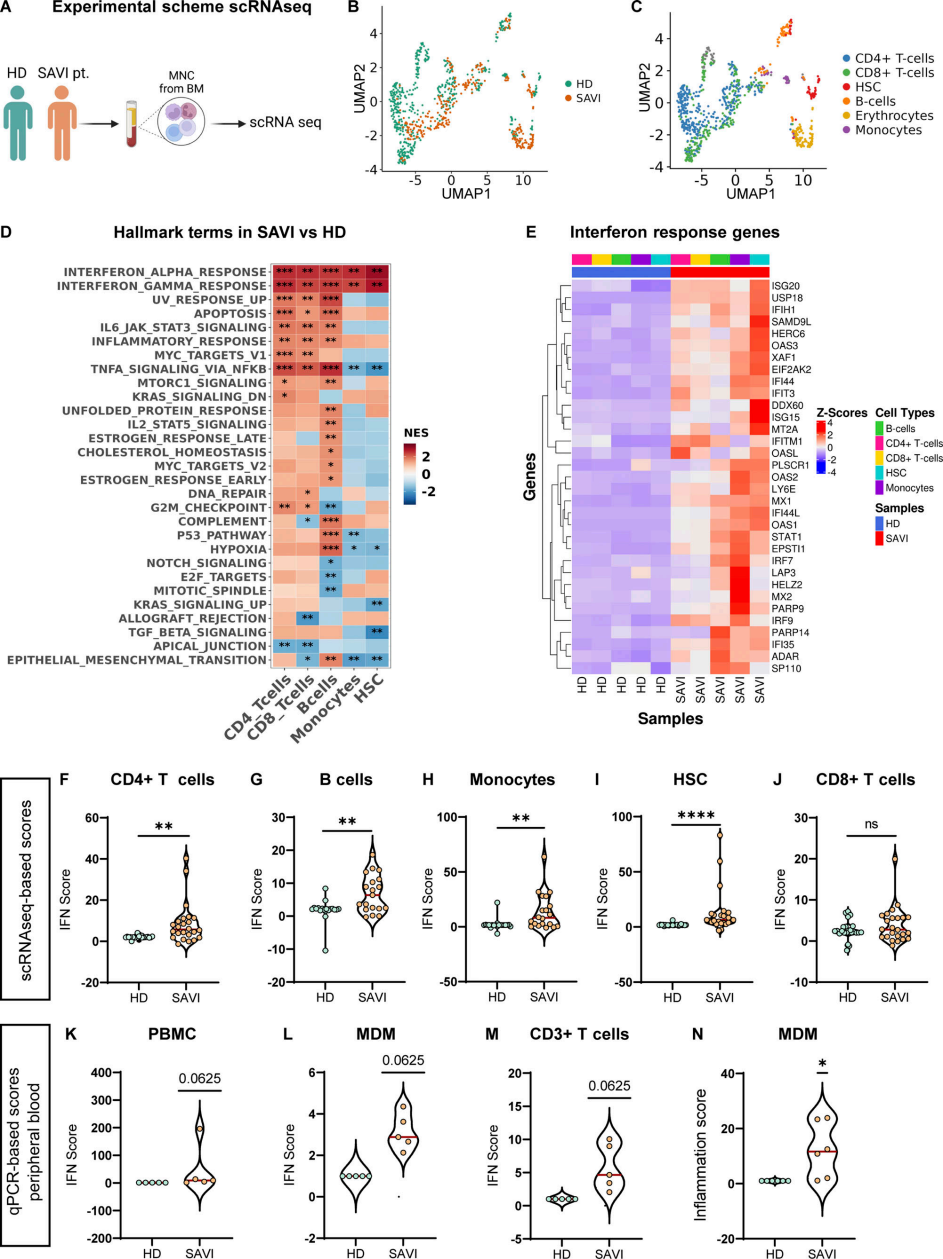
**Single-cell transcriptomic reveals spontaneous upregulation of type I IFN and inflammation in patient blood cells. (A)** Experimental scheme (created with https://BioRender.com) of the scRNAseq experiment performed on MNC from a BM sample of the SAVI patient and a pediatric HD control (*n* = 1 experiment). **(B and C)** UMAP plots showing cells from scRNAseq data of HD and SAVI samples. Cells are annotated by sample type (B) or according to the identified cell types (C). **(D)** Heatmap visualizing the enriched GSEA terms in the identified cell types against the Hallmark gene set (Molecular Signatures Database). GSEA was performed on logFC reranked gene lists obtained from SAVI gene expression compared with HD within each cell type (NES: normalized enrichment score; weighted Kolmogorov–Smirnov test with Benjamini-Hochberg adjusted P values; *, adjusted P < 0.05; **, adjusted P < 0.01; ***, adjusted P < 0.001). **(E)** Heatmap showing the expression level of genes belonging to the IFNα/γ pathways in the identified cell types of HD and SAVI samples. Gene expression, in rows, was row-scaled (z-scores) for visualization. **(F–J)** Violin plots showing the distribution of IFN scores from scRNAseq data in the identified cell populations. Scores were calculated from the expression of 28 ISGs in SAVI patient compared with HD control (Mann–Whitney test; **, P < 0.01; ****, P < 0.0001; ns = non-significant). **(K–M)** Violin plots showing the distribution of IFN scores measured in the indicated cell populations from qRT-PCR quantification of the median FC of five ISGs in SAVI patient compared with HD control (*n* = 3 independent experiments; Mann–Whitney test; P value numbers are shown). **(N)** Violin plots showing the distribution of the inflammation score measured in MDM from qRT-PCR quantification of the median FC of six IRGs in SAVI patients compared with HD control (*n* = 3 independent experiments; Mann–Whitney test; *, P < 0.05).

**Figure S1.**
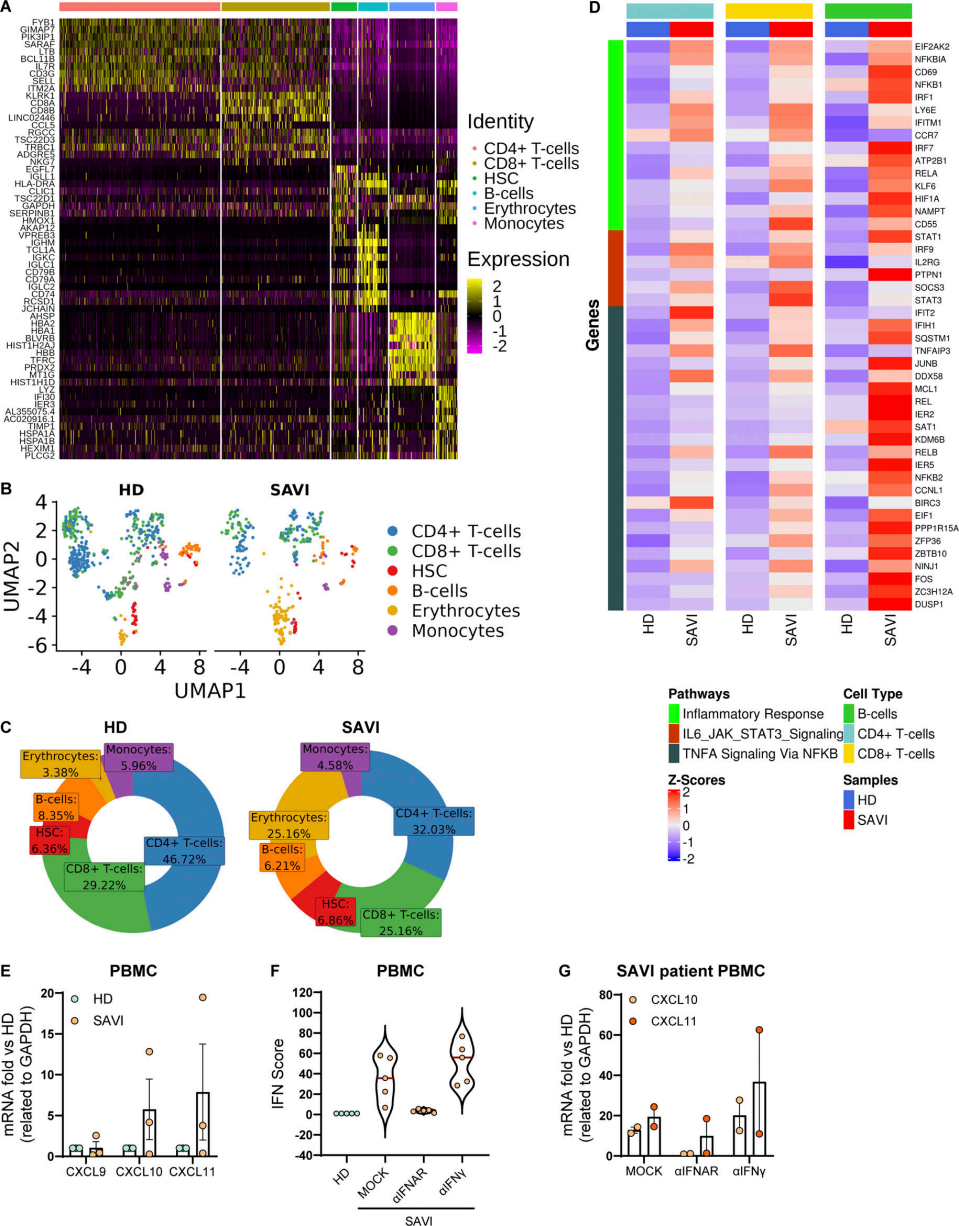
**Spontaneous activation of multiple inflammatory pathways identified by scRNAseq in SAVI patient blood cells. (A)** Hierarchically clustered average gene expression heatmap for genes overexpressed across the different cell types grouped according to the Seurat classification. Yellow, high expression; purple, low expression. Scaled-in normalized gene expression data are shown. **(B)** Cell type–resolved UMAPs showing cells from scRNAseq data of HD and SAVI samples side by side. **(C)** Donut plots showing the different cell type percentages quantified from single-cell data in HD and SAVI samples. **(D)** Heatmaps showing the expression of genes from the different GSEA terms identified in the indicated cell population between HD and SAVI. Gene expression, in rows, was row-scaled (z-scores) for visualization. **(E)** The expression of different IFNγ-induced cytokines was measured by RT-qPCR in the SAVI patient and HD PBMC and expressed as fold versus HD, normalized to the GAPDH housekeeping gene (mean ± SD; *n* = 3 independent experiments). **(F and G)** The distribution of the IFN score (F) and the expression of IFNγ-induced cytokines (G) was evaluated in SAVI patient PBMC after 24 h treatment with a neutralizing antibody targeting the αIFNAR or a neutralizing antibody against the IFNγ (αIFNγ) (each dot in Fig. S1 F represents one ISG calculated from one independent experiment; in Fig. S1 G technical duplicates from one independent experiment are shown).

Given the genetic diagnosis of SAVI disease, we hypothesized that the substitution of a serine for a phenylalanine in position 269 would lead to constitutive STING activation, resulting in the upregulation of inflammation and type I IFN responses, as previously reported for other SAVI-causing mutations (Wang et al., 2021; Fremond et al., 2021; Crow and Stetson, 2022). To investigate this, we performed single-cell RNA sequencing (scRNAseq) to simultaneously analyze the transcriptome of different blood cell populations from a bone marrow (BM) sample of the SAVI patient collected before the start of the immunosuppressive treatments. A pediatric healthy donor (HD) sample was used as a control (Fig. 2 A). Gene set enrichment analysis (GSEA) on marker genes allowed the identification of clusters of cells with gene signatures characteristic of CD4^+^ T cells, CD8^+^ T cells, B cells, monocytes, and hematopoietic stem cells (HSC) in both SAVI and HD (Fig. 2, B and C; and Fig. S1, A and B). In line with the reported T cell cytopenia, a reduced proportion of the total T cell compartment was observed in the SAVI BM compared with the healthy control, while minimal differences were present across the other cell subsets (Fig. S1 C). GSEA confirmed the significant upregulation of IFNα and IFNγ-related genes in patient-derived T cells, B cells, monocytes, and HSC in the absence of any exogenous trigger (Fig. 2, D and E). The activation of type I IFN was further confirmed by calculating the IFN score from the transcriptomic data using a set of 28 genes previously described (Kim et al., 2018) (Fig. 2, F–J). GSEA also confirmed the upregulation of several proinflammatory pathways, including TNFα via NF-κB, and JAK/STAT signaling in SAVI patient T and B cells (Fig. 2 D and Fig. S1 D), as well as significant modulation of other pathways related to DNA damage, proliferation, and cell death, previously reported to be dependent of STING activation (Wu et al., 2020; Hong et al., 2022; Fremond et al., 2021) (Fig. 2 D). The upregulation of IFN responses in the blood cells of the patient was confirmed during follow-up through the analysis of peripheral blood samples collected at different time points during steroids and/or JAK inhibitor treatment (Fig. 1 A). Despite the treatment, the IFN score, calculated by qRT-PCR of a selected set of genes (Lambers et al., 2019; Rice et al., 2013, 2018; Tungler et al., 2016), resulted higher in the patient compared with HD, in both total peripheral blood mononuclear cells (PBMC), isolated CD3^+^ T cells, and monocyte-derived macrophages (MDM) (Fig. 2, K–M). MDM showed also a higher inflammation score (Fig. 2 N) calculated on the expression of five different proinflammatory genes (Rice et al., 2013, 2018; Tungler et al., 2016; Giordano et al., 2022). Despite the lack of pretreatment data, the persistence of an IFN signature in the patient cells is in line with previous reports where baricitinib treatment demonstrated efficacy in reducing clinical manifestations in other SAVI patients, although incomplete inhibition of type I IFN signaling was observed (Sanchez et al., 2018; Fremond et al., 2016). The ISGs considered for the IFN score can be upregulated by both type I and type II IFNs. Given that IFNγ-related genes were among upregulated terms in the scRNAseq dataset and a role for type II IFN has been demonstrated in SAVI mouse models (Stinson et al., 2022), we further dissected the contribution of IFNγ to the SAVI phenotype. We first checked the expression of known IFNγ-inducible genes in patient PBMC collected at different time points. Although some variability was observed across time points, CXCL9, CXCL10, and CXCL11 resulted in overall upregulation compared with HD controls (Fig. S1 E). We next verified the impact of neutralizing antibodies against the IFNα receptor (αIFNAR) or the IFNγ on both the ISG signature and the expression of CXCL genes. Both were significantly impacted by the blockade of IFNα- but not IFNγ-mediated signaling, suggesting that type I rather than type II IFN drives these gene signatures in SAVI cells (Fig. S1, F and G).

Taken together, these data establish that type I IFN and several proinflammatory signaling cascades are spontaneously activated in immune cells and HSC carrying this novel *STING* variant causing SAVI disease, and they confirm the T cell lymphopenia typically observed in SAVI patients.

### STING F269S expression in immune cell lines recapitulates the SAVI patient phenotype

**Figure S2.**
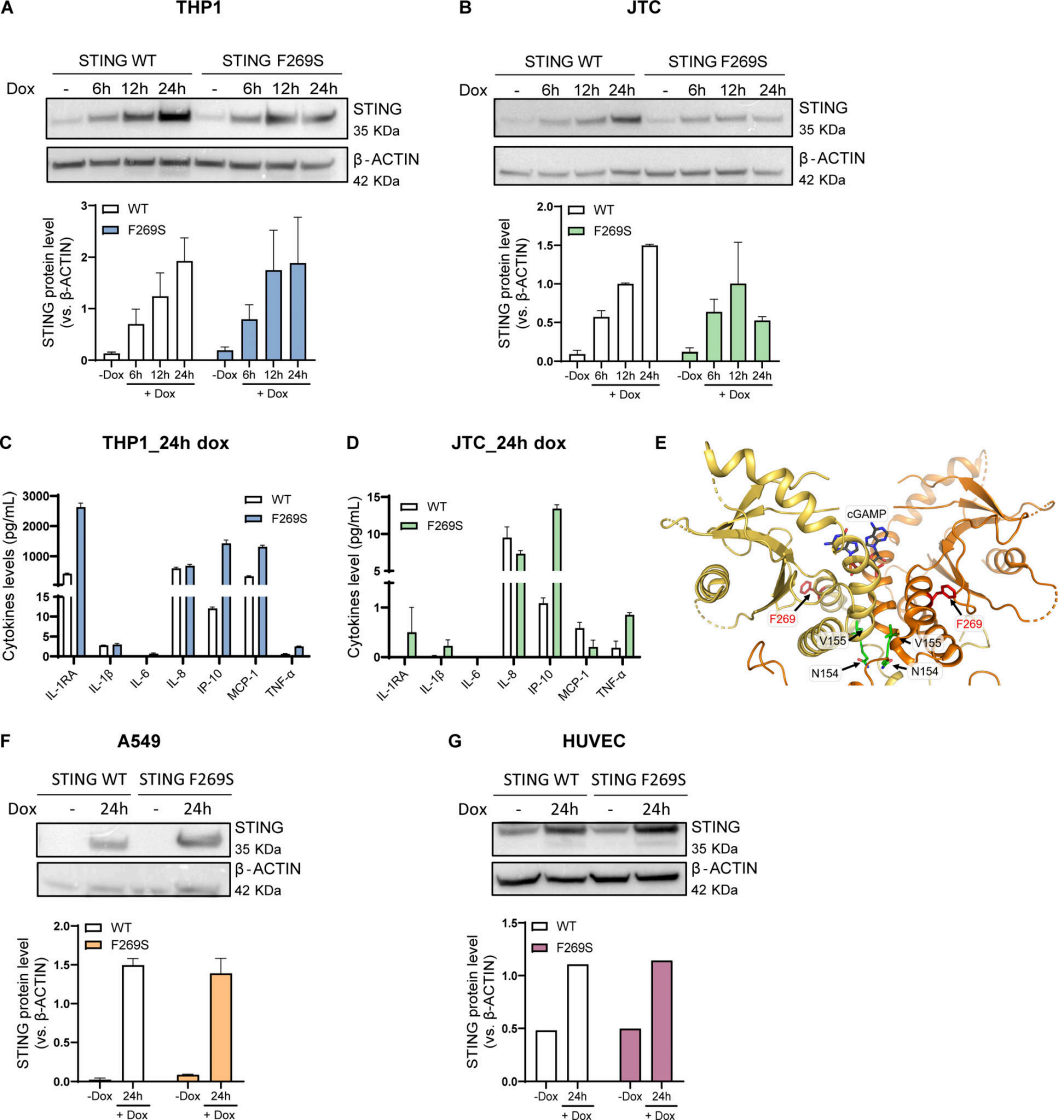
**Characterization of SAVI (STING F269S) cell lines. (A and B)** STING WT and STING F269S protein levels were evaluated in THP1 (A) and JTC (B) by western blot at different time points after dox exposure and quantified using ImageJ (mean ± SEM; *n* = 2 independent experiments; one representative blot is shown). **(C and D)** The levels of the indicated cytokines were measured in the supernatant of THP1 (C) and JTC (D) 24 h after dox-induced expression of STING WT or STING F269S by MSD-based assay (mean ± SEM; *n* = 1 experiment in technical duplicate). **(E)** Cartoon representation of the soluble portion of dimeric human STING, with cGAMP modeled in its binding site (gray sticks) based on the observed conformation adopted by the ligand in cryo-EM structures of the chick homolog. F269 is shown with red sticks, and interface residues involved in SAVI N154 and V155 are shown with green sticks. **(F and G)** STING WT and STING F269S protein levels were evaluated in A549 (F) and HUVEC (G) by western blot at 24 h after dox exposure and quantified using ImageJ (mean ± SEM; *n* = 1–2 independent experiments; one representative blot is shown). Source data are available for this figure: SourceData FS2.

**Figure 3.**
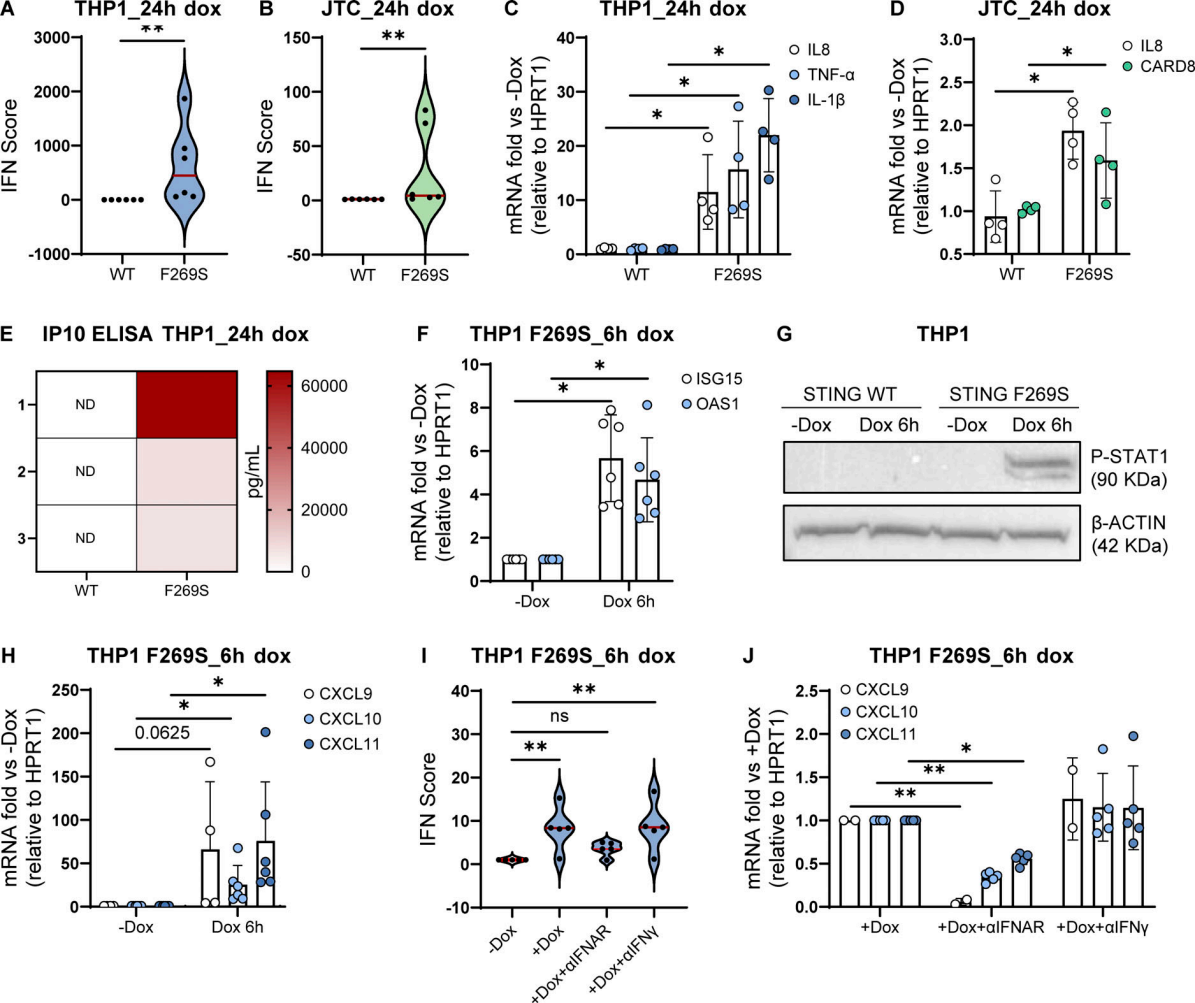
**STING F269S expression in immune cell lines recapitulates the SAVI patient phenotype. (A and B)** Violin plots showing the distribution of the IFN score in THP1 (A) and JTC (B) from qRT-PCR quantification of the median FC of six ISGs 24 h after dox-induced expression of STING WT or STING F269S. Each dot represents one ISG (*n* = 3 independent experiments; Mann–Whitney test; **, P < 0.01). **(C and D)** The expression of different inflammatory genes was measured by RT-qPCR 24 h after dox-induced expression of STING WT or STING F269S in THP1 (C) and JTC (D) and expressed as fold versus −dox, normalized to the HPRT1 housekeeping gene (mean ± SD; *n* = 4 independent experiments; Mann–Whitney test; *, P < 0.05). **(E)** IP10 levels were measured by ELISA in the supernatant of THP1 24 h after dox induced expression of STING WT or STING F269S (*n* = 3 independent experiments, ND = not detected). **(F)** ISGs levels were measured by RT-qPCR 6 h after dox-induced expression of STING F269S in THP1 and expressed as fold versus −dox, normalized to the HPRT1 housekeeping gene (mean ± SD; *n* = 6 independent experiments; one sample Wilcoxon test; *, P < 0.05). **(G)** STAT1 phosphorylation was evaluated by western blot in THP1 6 h after dox-induced expression of STING WT or STING F269S. β-ACTIN was used as loading control (*n* = 2 independent experiments; one representative blot is shown). **(H)** IFNγ-inducible genes were measured by RT-qPCR 6 h after dox–induced expression of STING F269S in THP1 and expressed as fold versus −dox, normalized to the HPRT1 housekeeping gene (mean ± SD; *n* = 4/6 independent experiments; one sample Wilcoxon test; *, P < 0.05). **(I)** Violin plots showing the distribution of the IFN score in THP1 F269S 6 h after treatment with dox ± αIFNAR or αIFNγ. Each dot represents one ISG (*n* = 4 independent experiments; two-way ANOVA with Dunnett’s multiple comparisons; **, P < 0.01; ns = not significant). **(J)** IFNγ-inducible genes were measured by RT-qPCR in THP1 F269S 6 h after treatment with dox ± αIFNAR or αIFNγ and expressed as fold versus +dox, normalized to the HPRT1 housekeeping gene (mean ± SD, *n* = 5 independent experiments; two-way ANOVA with Tukey’s multiple comparisons; *, P < 0.05; **, P < 0.01). Source data are available for this figure: SourceData F3.

**Figure 4.**
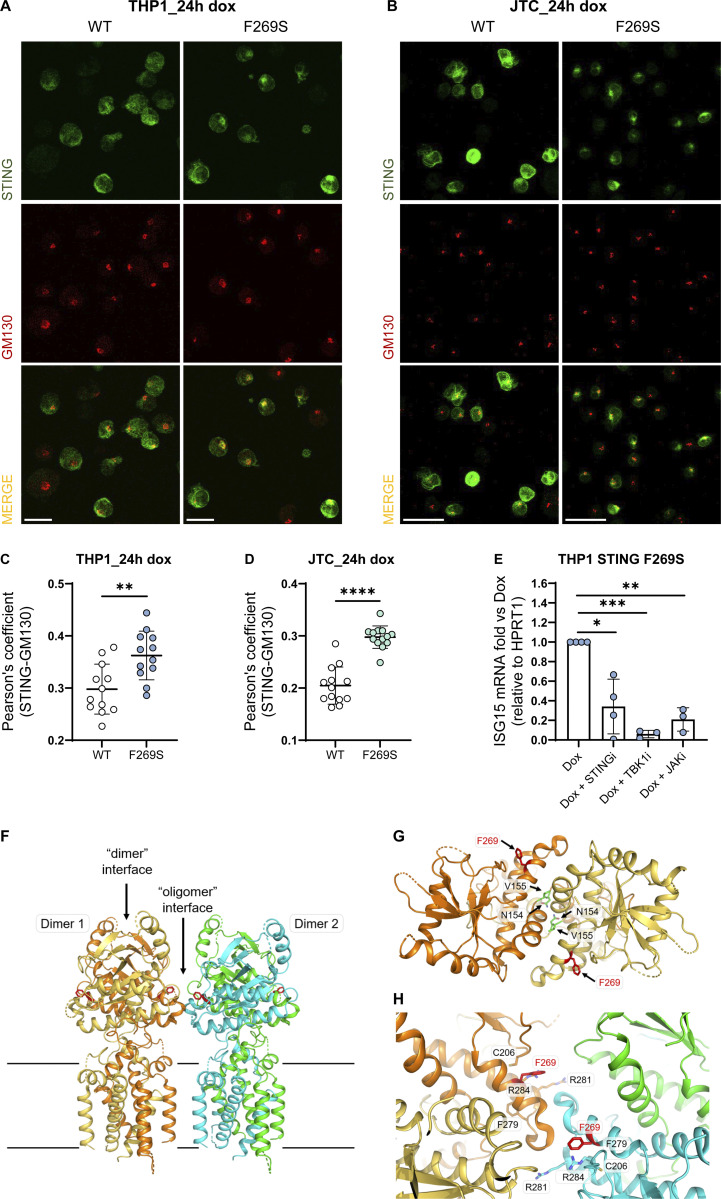
**STING F269S spontaneously translocates to the Golgi. (A and B)** Representative IF images acquired using TCS SP5 Leica confocal microscope, 40× with oil on THP1 (A) or JTC (B) 24 h after dox induced expression of STING WT or STING F269S. Colocalization (yellow area) of STING (green) with the Golgi marker GM130 (red) was evaluated (scale bar, 20 μm). **(C and D)** Quantification of Pearson’s colocalization coefficient between STING and the Golgi marker GM130 was performed on THP1 (C) and JTC (D) 24 h after dox-induced expression of STING WT or STING F269S (mean ± SD; *n* = 12–13 images acquired from two independent experiments; Mann–Whitney test; **, P < 0.01; ****, P < 0.0001). **(E)** THP1 STING F269S were treated with dox and the indicated drugs for 24 h, and the expression of ISG15 was measured by RT-qPCR and expressed as fold versus dox, normalized to the HPRT1 housekeeping gene (mean ± SD; *n* = 3–4 independent experiments; one sample *t* test; **, P < 0.01; ***, P < 0.001; ns = not significant). **(F)** Molecular mapping of the F269S mutation on available STING three-dimensional structures. Cartoon representation of the human STING oligomeric ensemble, modelled based on the cryo-EM structure of chick STING oligomers. Each STING monomer is shown using a different color. The black lines indicate the boundaries of the transmembrane region. **(G)** Residue F269 (displayed as red sticks) localizes far from the STING dimer interface, characterized by the extensively studied mutations involving residues N154 and V155 (displayed as green sticks). **(H)** F269 localizes at the interface identified in STING oligomeric structures, resulting in contacts involving two distinct STING dimers in close proximity to previously characterized residues C206, F279, R281, and R284.

To gain deeper insights into the mechanism and consequences of the STING F269S mutation in human immune cells, we generated cell line models by introducing the STING F269S protein in human promonocytic and lymphoid cell lines. Cells were transduced with a lentiviral vector (LV) to enable controlled doxycycline (dox)-inducible expression of either STING WT or STING F269S mutant (Fig. S2, A and B). In Jurkat T cells (JTC), the levels of the STING F269S protein tended to decrease at later time points (Fig. S2 B), suggesting an increased degradation of the mutated protein compared with the WT, in line with its sustained activation and trafficking (Balka et al., 2023). Overexpression of STING F269S in both THP1 and JTC induced a strong upregulation of different ISGs at 24 h, leading to higher IFN scores compared with STING WT expressing cells (Fig. 3, A and B). We next evaluated the inflammatory profile of both SAVI cell line models. STING F269S-expressing cells exhibited increased expression of proinflammatory genes (Fig. 3, C and D) and increased secretion of different cytokines and chemokines compared with STING WT cells (Fig. S2, C and D). IP-10, also known as CXCL10, was highly secreted in both SAVI models (Fig. S2, C and D), as confirmed by ELISA assay in THP1 cells (Fig. 3 E). ISGs upregulation was observed as early as 6 h (Fig. 3 F) concomitant with early STAT1 phosphorylation (Fig. 3 G), suggesting that low levels of STING F269S are sufficient to mediate activation of type I IFN. Similarly, IFNγ-inducible genes resulted strongly upregulated in SAVI THP1 cells at 6 h (Fig. 3 H). In line with data from patient PBMC, both the ISG signature and the expression of CXCL genes appeared mainly dependent on IFNα secretion, with no impact observed when inhibiting IFNγ (Fig. 3, I and J), further confirming a main contribution of type I versus type II IFN to the SAVI signature. To molecularly confirm that the increased activation of type I IFN responses and inflammation was directly linked to STING activation, we examined its localization. An increased colocalization of the mutated protein with the Golgi marker GM130 was observed in both THP1 and JTC (Fig. 4, A–D), confirming spontaneous STING translocation from the ER to the Golgi in the absence of ligands. It has been shown that inhibition of palmitoylation suppresses STING signaling in the V147L and N154S STING variants (Mukai et al., 2016). We thus assessed the impact of the STING palmitoylation inhibitor H151 (Haag et al., 2018) on the activation of the STING F269S variant, along with inhibitors acting at different steps of the STING signaling cascade. Inhibition of the downstream TBK1 and JAK kinases led to complete abrogation of type I IFN response (Fig. 4 E), while a 40–90% reduction was observed when targeting STING with the H151 inhibitor. To get more insight into the mechanism of STING autoactivation, we modeled the de novo mutation F269S on publicly available STING structures. F269 localizes in the ligand-binding domain (LBD), outside the dimer interface, where the most common N154 and V155 variants localize (Saldanha et al., 2018; Lin et al., 2020; Keskitalo et al., 2019) (Fig. 4, F and G), and distant from the cGAMP binding site (Fig. S2 E). F269 is in close proximity to R281 and R284 at the dimer–dimer contact sites observed during STING oligomerization (Liu et al., 2023b; Shang et al., 2019) (Fig. 4, F–H). Residues located in this region have been suggested to maintain STING inactive through side-by-side packing. As a result, the conversion of F269 to S269 may affect the stability of the interface, relieving the autoinhibition of STING oligomerization, thereby promoting its activation (Lin et al., 2020; Ergun et al., 2019; Konno et al., 2018; Liu et al., 2023b; Shang et al., 2019).

Overall, our data confirm the pathological nature of the STING F269S variant as a constitutively active variant that spontaneously translocates to the Golgi, thus driving IFN responses and inflammation in the absence of exogenous triggers.

### The STING F269S mutation associates with T cell cytopenia and lack of memory T cell phenotype

**Figure 5.**
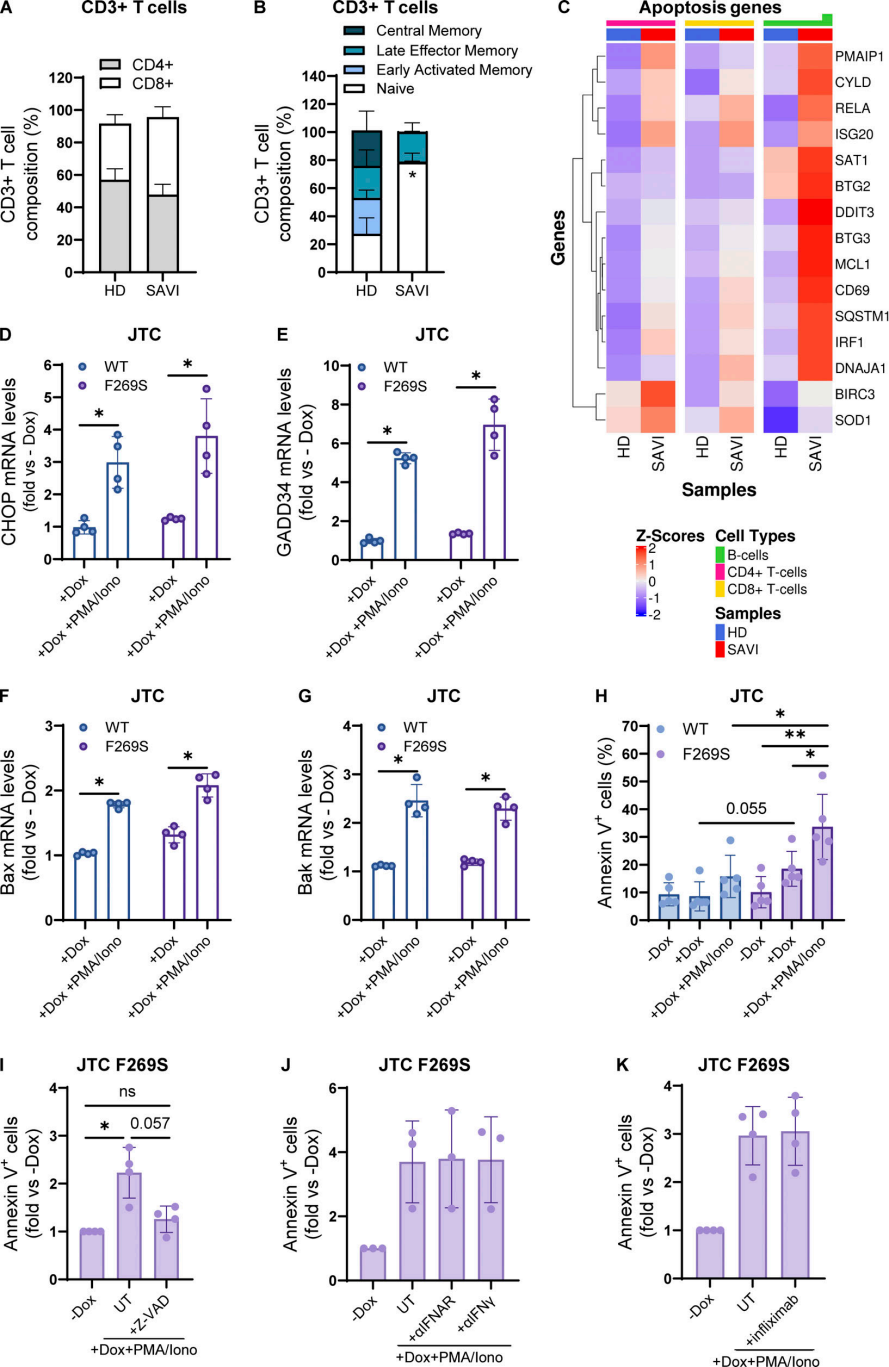
**The STING F269S mutation is associated with T cell cytopenia and lack of memory T cell phenotype. (A)** The relative percentages of CD4^+^ and CD8^+^ T cells was evaluated in HD and SAVI patient CD3^+^ T cells by FACS at day 5 after in vitro antiCD3/CD28 beads stimulation (mean ± SEM; *n* = 3 independent experiments). **(B)** T cell subpopulation composition was evaluated in HD and SAVI patient CD3^+^ T cells by FACS at day 5 after in vitro antiCD3/CD28 beads stimulation (mean ± SEM; *n* = 3 independent experiments; unpaired *t* test; *, P < 0.05). **(C)** Heatmap showing the expression level of genes belonging to the apoptosis pathway in HD and SAVI B cells, CD4^+^ and CD8^+^ T cells from scRNAseq data. Gene expression, in rows, was row-scaled (z-scores) for visualization. **(D and E)** JTC were treated with dox for 24 h, washed, and stimulated for an additional 24 h with PMA/ionomycin. The expression of the UPR genes CHOP (D) and GADD34 (E) was measured by RT-qPCR and expressed as fold versus −dox, normalized to the HPRT1 housekeeping gene (mean ± SD; *n* = 4 independent experiments; Mann–Whitney test; *, P < 0.05). **(F and G)** JTC were treated with dox for 24 h, washed, and stimulated for additional 24 h with PMA/ionomycin. The expression of the pro apoptotic genes Bax (F) and Bak (G) was measured by RT-qPCR and expressed as fold versus −dox, normalized to the HPRT1 housekeeping gene (mean ± SD; *n* = 4 independent experiments; Mann–Whitney test; *, P < 0.05). **(H)** JTC were treated with dox for 24 h, washed, and stimulated for an additional 24 h with PMA/ionomycin. The percentage of apoptotic cells was evaluated by FACS (mean ± SD; *n* = 5 independent experiments; Mann–Whitney test; *, P < 0.05; **, P < 0.01). **(I)** JTC were treated with dox for 24 h, washed, and stimulated for an additional 24 h with PMA/ionomycin in the presence or not of the pan caspase inhibitor Z-VAD. The percentage of apoptotic cells was evaluated by FACS and expressed in fold versus −dox (mean ± SD; *n* = 4 independent experiments; one sample Wilcoxon test versus −dox = 1; *, P < 0.05; ns = not significant; Mann–Whitney test between −/+Z-VAD comparison; P value number is shown). **(J and K)** JTC were treated with dox for 24 h, washed, and stimulated for additional 24 h with PMA/ionomycin in the presence or not of the indicated inhibitors. The percentage of apoptotic cells was evaluated by FACS and expressed in fold versus −dox (mean ± SD; *n* = 3 independent experiments for J; *n* = 4 independent experiments for K).

**Figure S3.**
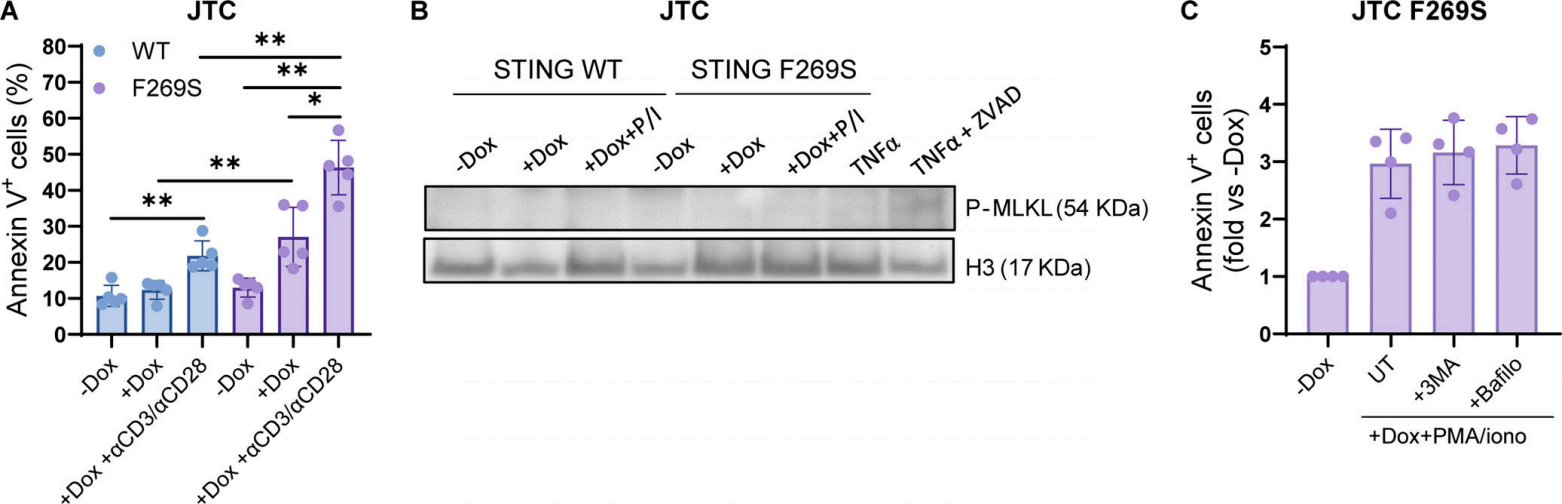
**The STING F269S mutation is associated with T cell cytopenia and lack of memory T cell phenotype. (A)** JTC were treated with dox for 24 h, washed, and stimulated for additional 24 h with anti-CD3, anti-CD28 antibodies. The percentage of apoptotic cells was evaluated by FACS (mean ± SD; *n* = 5 independent experiments; Mann–Whitney test; *, P < 0.05; **, P < 0.01). **(B)** MLKL phosphorylation was evaluated by western blot in JTC treated with dox for 24 h and stimulated for additional 24 h with PMA/ionomycin (P/I). JTC treated with TNFα ± ZVAD were added as the positive control. H3 was used as loading control (*n* = 2 independent experiments; one representative blot is shown). **(C)** JTC were treated with dox for 24 h, washed, and stimulated for additional 24 h with PMA/ionomycin in the presence or not of the indicated autophagy inhibitors. The percentage of apoptotic cells was evaluated by FACS and expressed in fold versus –dox (mean ± SD; *n* = 4 independent experiments). Source data are available for this figure: SourceData FS3.

Patients with GOF mutations in STING frequently display lymphopenia (Cerboni et al., 2017; Liu et al., 2014) and intrinsic defects in anti-CD3/CD28 T cell proliferation, as observed in our patient (Table 1). The reduction of the T cell memory phenotype observed in the patient at onset was confirmed during follow-up through the analysis of peripheral blood T cells at different time points during pharmacological treatment. While the CD4^+^/CD8^+^ T cell ratio was similar to HD controls (Fig. 5 A), a higher prevalence of the T naïve compartment was observed in SAVI T cells, which almost completely lacked the memory phenotype (Fig. 5 B). It has been shown that chronic activation of STING triggers ER stress and the unfolded protein response (UPR), priming T cells to become hyperresponsive to TCR signaling and promoting cell death (Wu et al., 2019). Interestingly, apoptosis and UPR genes were among upregulated terms in patient CD4^+^, CD8^+^ T cells, and B cells (Fig. 2 D and Fig. 5 C), prompting us to investigate whether a similar cell death mechanism occurs in STING F269S mutated cells. To dissect the cell-intrinsic effect of STING F269S activation in human T cells, we exploited the previously generated JTC SAVI model. Cells were first exposed to dox for 24 h to induce STING expression, washed, and stimulated with PMA+ionomycin to mimic TCR activation for an additional 24 h. While TCR-mimicking stimulation upregulated UPR response (Fig. 5, D and E) and pro-apoptotic gene expression (Fig. 5, F and G) to a similar extent in STING WT and F269S cells, a robust increase in apoptosis was observed exclusively in STING F269S JTC (Fig. 5 H). These data were confirmed also upon direct TCR stimulation with anti-CD3/anti-CD28 antibodies (Fig. S3 A). Treatment of STING F269S JTC with the pan-caspase inhibitor Z-VAD during PMA/ionomycin stimulation strongly prevented the induction of apoptosis (Fig. 5 I) and absence of MLKL phosphorylation after PMA/ionomycin stimulation suggested no occurrence of necroptosis (Fig. S3 B). To investigate the role of cytokine secretion on the SAVI JTC death, we treated the cells with αIFNAR, αIFNγ, or infliximab to block TNFα signaling. Neither inhibitor prevented the induction of apoptosis (Fig. 5, J and K), suggesting IFN- and TNF-independent cell death mechanisms and indicating that apoptosis may occur independently from paracrine signaling. Given that STING plays an important role in the induction and regulation of autophagy under different stress conditions (Zhang et al., 2021), we next evaluated the autophagy-dependent cell death mechanism. Neither 3-methyladenine (3-MA) nor bafilomycin A1 treatment protected cells from apoptosis (Fig. S3 C), suggesting that other mechanisms prime the SAVI cell for cell death.

Overall, these data suggest that while WT cells can tolerate TCR-mediated activation stress, STING F269S intrinsically primes cells for apoptotic cell death when exposed to TCR signaling through mechanisms that are independent of cytokine secretion and autophagy. Of note, a trend in increased apoptosis was observed in STING F269S cells even in the absence of TCR stimulation, highlighting a TCR-independent apoptotic phenotype linked to STING activation.

Taken together, our data show a lack of T cell memory phenotype in our SAVI patient and highlight STING-mediated cell death as a potentially relevant pathway for the control of T cell numbers and the establishment of the memory subset in SAVI patients.

### STING F269S immune cell secretome promotes endothelial cell activation and damage

**Figure 6.**
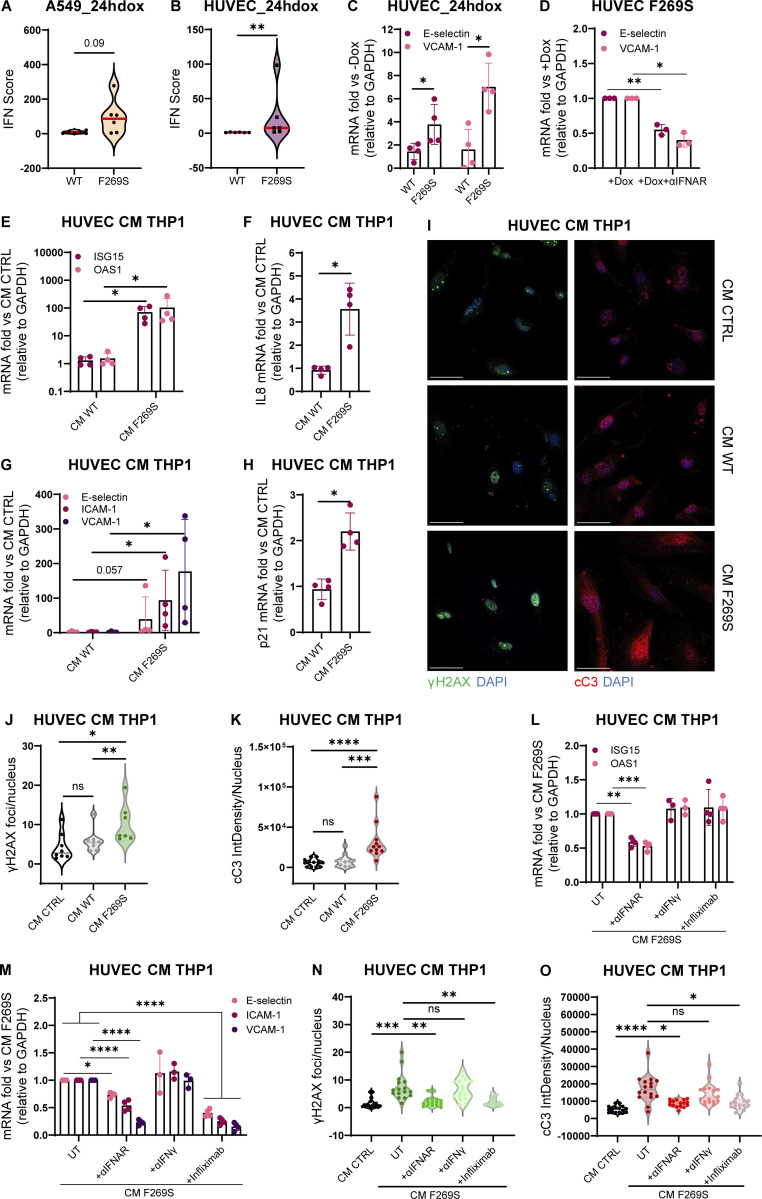
**STING F269S immune cell secretome promotes endothelial cells activation and damage. (A and B)** Violin plots showing the distribution of the IFN score in A549 (A) and HUVEC (B) from qRT-PCR quantification of the median FC of six ISGs 24 h after dox induced expression of STING WT or STING F269S. Each dot represents one ISG (*n* = 3 independent experiments; Mann–Whitney test; **, P < 0.01). **(C)** The expression of adhesion molecules was evaluated in HUVEC 24 h after dox induced expression of STING WT or STING F269S by qRT-PCR and expressed as fold versus −dox, normalized to the GAPDH housekeeping gene (mean ± SD; *n* = 4 independent experiments; Mann–Whitney test; *, P < 0.05). **(D)** The expression of adhesion molecules was evaluated in HUVEC F269S 24 h after dox treatment ± αIFNAR and expressed as fold versus +dox, normalized to the GAPDH housekeeping gene (mean ± SD; *n* = 3 independent experiments; one sample *t* test; *, P < 0.05; **, P < 0.01). **(E–H)** The expression of ISGs (E), inflammatory gene (F), endothelial adhesion markers (G), and p21 DNA damage marker (H) was measured by RT-qPCR in HUVEC cells 24 h after exposure to CM from THP1 STING WT or STING F269S and expressed as fold versus control medium (CM CTRL), normalized to the GAPDH housekeeping gene (mean ± SD; *n* = 4 independent experiments; Mann–Whitney test; *, P < 0.05). **(I)** Representative IF images acquired using Olympus FluoVIEW 3000 RS confocal microscope, 60× with oil, on HUVEC cells 24 h after exposure to CM from THP1 STING WT or STING F269S stained for γH2AX or cC3 (scale bar, 50 μm). **(J and K)** The number of γH2AX foci (J) and integrated density of cC3 signal (K) were quantified by ImageJ (*n* = 7–8 images from two independent experiments for γH2AX; *n* = 11–12 images from three independent experiments for cC3; Mann–Whitney test; *, P < 0.05; **, P < 0.01; ***, P < 0.001; ****, P < 0.0001; ns = not significant). **(L and M)** CM from THP1 STING F269S were added to HUVEC cells in combination with the indicated drugs for 24 h. The expression of ISGs (L) and endothelial adhesion markers (M) was measured by RT-qPCR in HUVEC cells and expressed as fold versus CM F269S UT, normalized to the GAPDH housekeeping gene (mean ± SD; *n* = 3–4 independent experiments; two-way ANOVA with Tukey’s multiple comparisons; *, P < 0.05; **, P < 0.01; ***, P < 0.001; ****, P < 0.0001). **(N and O)** HUVEC cells were exposed for 24 h to CM from THP1 STING F269S in combination with the indicated drugs or control CM. The number of γH2AX foci (N) and the integrated density of cC3 signal (O) were quantified by ImageJ (*n* = 16 images from 2 independent experiments; Kruskal–Wallis test with Dunn’s multiple comparisons; *, P < 0.05; **, P < 0.01; ***, P < 0.001; ****, P < 0.0001; ns = not significant).

**Figure S4.**
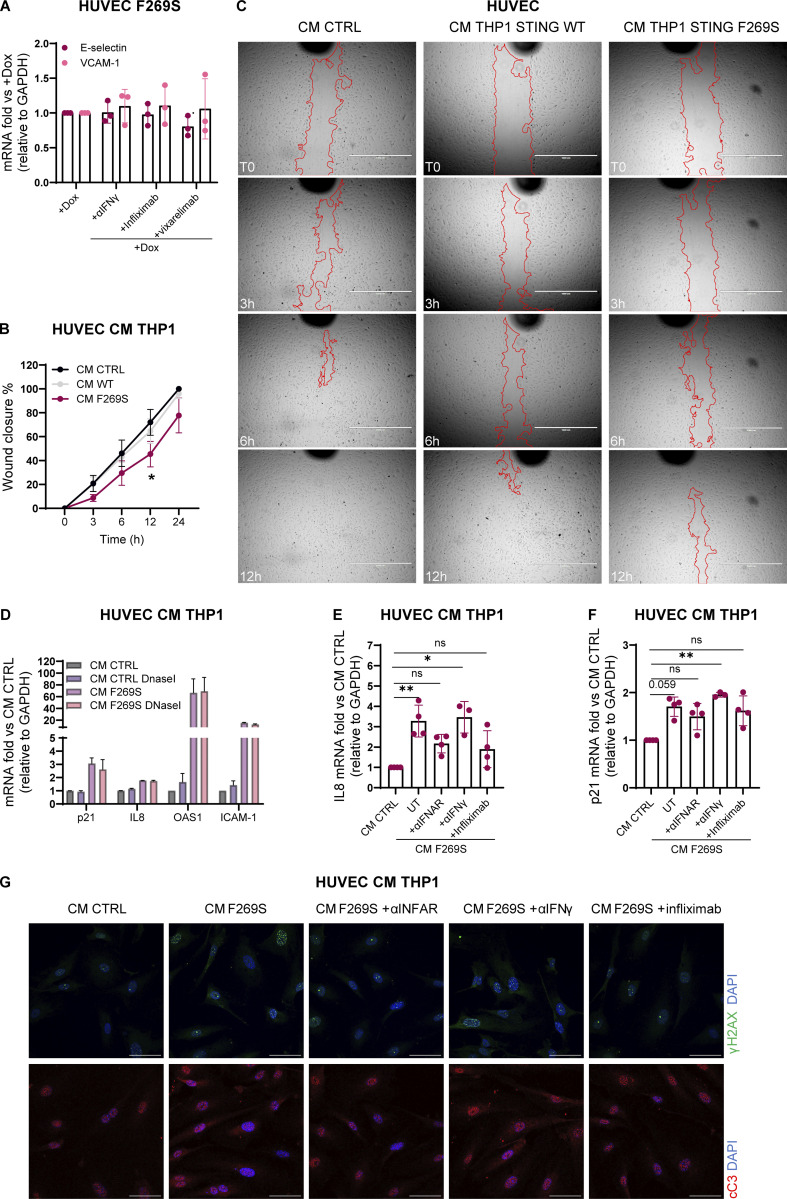
**Impact of SAVI immune cells secretome on endothelial cells. (A)** The expression of adhesion molecules was evaluated in HUVEC F269S 24 h after dox treatment in the presence or not of the indicated drugs and expressed as fold versus +dox, normalized to the GAPDH housekeeping gene (mean ± SD; *n* = 3 independent experiments). **(B)** HUVEC cells were exposed for 24 h to CM from THP1 STING WT or STING F269S. A scratch wound assay was then performed, and the percentage of wound closure was evaluated at indicated time points after the scratch (mean ± SD; *n* = 5 independent experiments; two-way ANOVA versus CM CTRL; *, P < 0.05). **(C)** Representative bright-field images of scratch-wound closure from one of the experiments shown in Fig. S4 B monitored over time in HUVEC cells upon exposure to CM derived from THP1 STING WT, THP1 STING F269S, or to control CM (*n* = 1 of 5 independent experiments is shown; scale bar, 1,000 μm). **(D)** CM from THP1 STING F269S or control CM were treated for 30′ with DnaseI before addition to HUVEC cells. The expression of selected genes was measured by RT-qPCR in HUVEC cells 24 h after exposure to CM and expressed as fold versus CM CTRL, normalized to the GAPDH housekeeping gene (mean ± SD; *n* = 2 independent experiments). **(E and F)** CM from THP1 STING F269S were added to HUVEC cells in combination with the indicated drugs for 24 h. The expression of IL8 (E) and p21 (F) was measured by RT-qPCR in HUVEC cells and expressed as fold versus CM CTRL, normalized to the GAPDH housekeeping gene (mean ± SD; *n* = 3–4 independent experiments; Kruskal–Wallis test with Dunn’s multiple comparisons; *, P < 0.05; **, P < 0.01; ns = not significant). **(G)** Representative IF images were acquired using Olympus FluoVIEW 3000 RS confocal microscope, 60× with oil, on HUVEC cells 24 h after exposure to CM CTRL or CM from THP1 STING F269S and the indicated drugs, stained for γH2AX or cC3. Quantifications are shown in Fig. 6, N and O (scale bar, 50 μm).

While the role of STING is mainly studied in cells of hematopoietic origin, it has been shown that STING is expressed by non-hematopoietic cells, such as lung epithelial cells and endothelial cells (Gao et al., 2022; Digre and Lindskog, 2021; Tabula Muris Consortium, 2018; Liu et al., 2014). Given that among the clinical manifestations, ILD and vasculopathy are frequently observed in SAVI, we investigated the cell-intrinsic effect of STING F269S expression in these cell types to better dissect how they can contribute to inflammation and disease pathogenesis. We exploited the previously described inducible system to express STING F269S in A549, as a model of lung epithelial cells, and HUVEC, as a primary endothelial cell source (Fig. S2, F and G). Consistent with a potential role for both epithelial and endothelial cells in SAVI lung inflammation, expression of the mutant STING led to increased IFN scores, especially in HUVEC (Fig. 6, A and B). In addition, an increased expression of adhesion molecules, such as VCAM-1 and E-selectin, was observed in SAVI endothelial cells (Fig. 6 C). While targeting the αIFNAR strongly reduced the expression of adhesion molecules (Fig. 6 D), no effect was observed when cells were treated with αIFNγ, infliximab, or vixarelimab that targets oncostatin M receptor (Fig. S4 A). These results suggest that STING F269S expression induces endothelial activation through type I IFN signaling, potentially promoting interaction with immune cells. Since STING/IRF3-dependent IFN production by macrophages has been reported as a key driver of severe ANCA-associated vasculitis leading to endothelial damage, pulmonary hemorrhages, and lung dysfunction (Kessler et al., 2022), we sought to determine the impact of SAVI immune cells in further promoting endothelial activation and inflammation. For this purpose, HUVEC WT were treated for 24 h with conditioned media (CM) collected from THP1 cells 24 h after STING WT or F269S induction. The CM derived from SAVI THP1 induced a strong upregulation of ISGs (Fig. 6 E) and proinflammatory genes (Fig. 6 F) in HUVEC cells and a strong expression of adhesion molecules (Fig. 6 G). Interestingly, a slight increase in the expression of the DNA damage marker p21 was observed upon SAVI THP1 CM treatment (Fig. 6 H) with concomitant increase in phosphorylation of H2AX (Fig. 6, I and J), increased cleaved Caspase-3 signal (cC3) (Fig. 6, I and K), and reduced cell migration (Fig. S4, B and C), overall highlighting cytokine-mediated endothelial dysfunction, cell damage, and apoptosis. To exclude the contribution of nucleic acids derived from dead cells to the observed phenotypes, CM from THP1 STING F269S were treated with DNaseI for 30′ prior to the addition of HUVEC cells, with no impact observed on the upregulation of the previously described genes (Fig. S4 D). We next sought to determine the key mediators involved in endothelial responses. For this purpose, HUVEC cells were exposed to CM in combination with a neutralizing antibody targeting the αIFNAR or the IFNγ, or with infliximab to inhibit the TNFα cascade. While IFNγ blockade had no impact on endothelial activation and viability, type I IFNs were the main drivers of ISG upregulation in the HUVEC cells exposed to CM (Fig. 6 L), and both IFNα and TNFα contributed to the inflammatory profile (Fig. S4 E) and the upregulation of adhesion molecules (Fig. 6 M), with a minimal impact of both on p21 expression (Fig. S4 F). In agreement, lower CM-mediated toxicity was observed, as measured by H2AX histone phosphorylation (Fig. 6 N and Fig. S4 G) and cleaved Caspase-3 signal (Fig. 6 O and Fig. S4 G), when IFNα or TNFα signaling was prevented.

Together, these data show that expression of STING F269S in endothelial cells is sufficient to promote endothelial inflammation and activation that is further exacerbated by cytokines secreted by immune cells. While IFNα is the main cytokine contributing to increased ISG signatures, it is not the sole triggering endothelial response. We identified TNFα as another key driver of endothelial cell activation and apoptosis, independently from the type I IFN signature. Overall, these data suggest that both endothelial and immune cells contribute to SAVI inflammation and tissue damage in the absence of exogenous stimuli.

### SAVI patient cells are less susceptible to viral infection but hyporesponsive to exogenous immune priming

**Figure 7.**
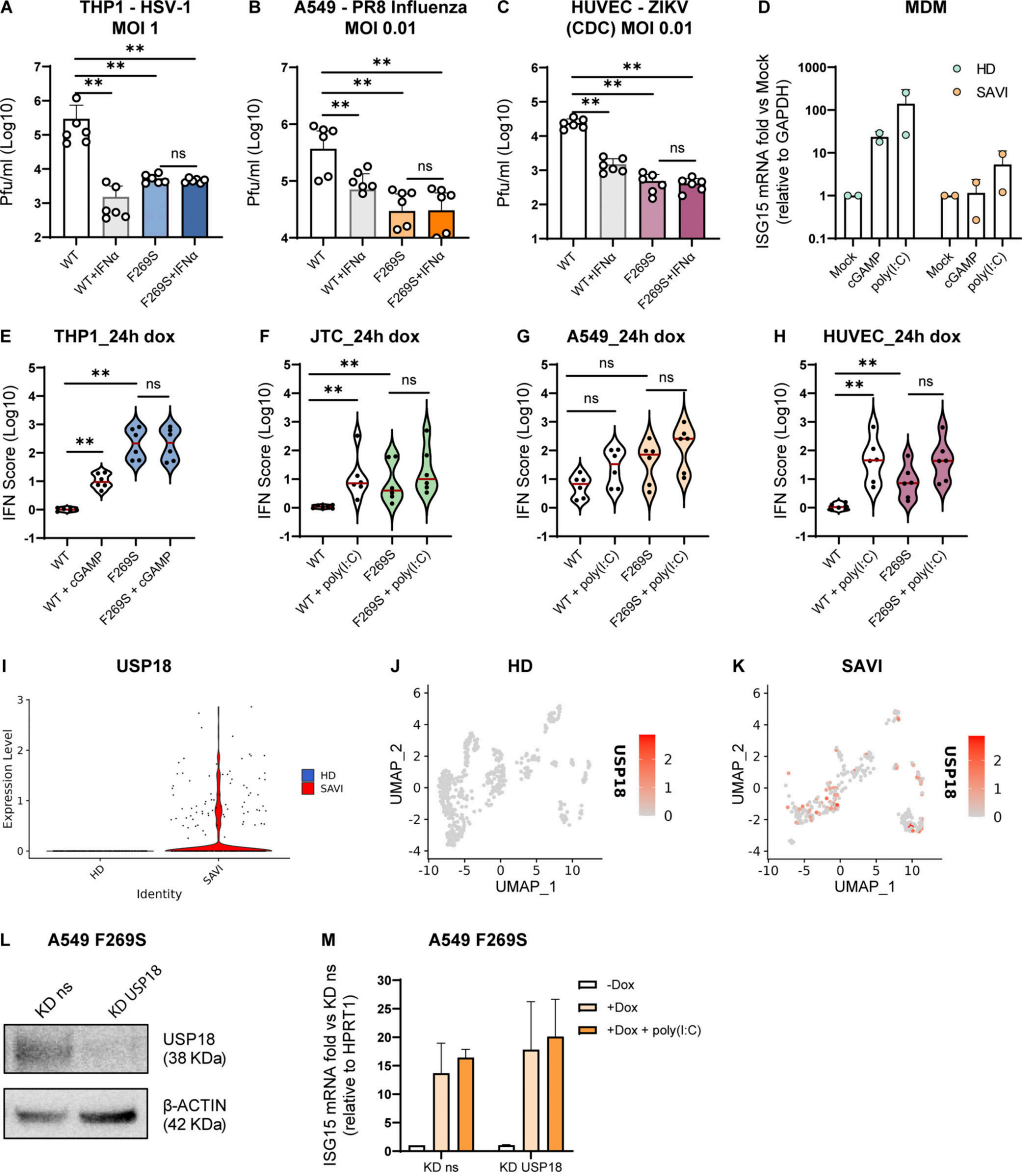
**SAVI patient cells are less susceptible to viral infection but hyporesponsive to exogenous immune priming. (A–C)** THP1 (A), A549 (B), and HUVEC (C) were prestimulated or not with IFNα for 16 h and then inoculated with the indicated infectious viruses. Viral supernatant was collected at 3 days after infection and titered on Vero cells (for HSV-1 and ZIKV) or MDCK cells (for IAV) (mean ± SD; *n* = 3 independent experiments; Mann–Whitney test; **, P < 0.01; ns = non-significant). **(D)** MDM from the SAVI patient and HD controls were stimulated with poly(I:C) or 2′3′cGAMP, and ISG15 level was measured at 24 h by qRT-PCR and expressed as fold versus mock, normalized to the GAPDH housekeeping gene (mean ± SD; *n* = 2 independent experiments). **(E–H)** Cells were treated with dox and stimulated with cGAMP or poly(I:C) for 24 h. The IFN score was calculated 24 h after dox administration in THP1 (E), JTC (F), A549 (G), and HUVEC (H) from qRT-PCR quantification of the median FC of six ISGs. Each dot represents one ISG (*n* = 3 independent experiments; Mann–Whitney test; **, P < 0.01; ns = not significant) (violin plots showing IFN score without stimulation are reported also in Fig. 3, A and B; and Fig. 6, A and B). **(I)** Violin plot showing the normalized expression level of USP18 from scRNAseq data in SAVI and HD samples. **(J and K)** UMAP showing the expression level and distribution of USP18 gene in HD (J) and SAVI (K) samples from scRNAseq data. **(L)** A549 STING F269S were transfected with siRNA targeting USP18 or a non-silencing (ns) siRNA control. Knock-down (KD) efficiency of USP18 was verified after 48 h by western blot. β-ACTIN was used as loading control (*n* = 2 independent experiments; one representative blot is shown). **(M)** A549 STING F269S were treated with dox and stimulated or not with poly(I:C) for 24 h. ISG15 level was measured by RT-qPCR and expressed as fold versus KD ns −dox, normalized to the HPRT1 housekeeping gene (mean ± SD; *n* = 3 independent experiments). Source data are available for this figure: SourceData F7.

**Figure S5.**
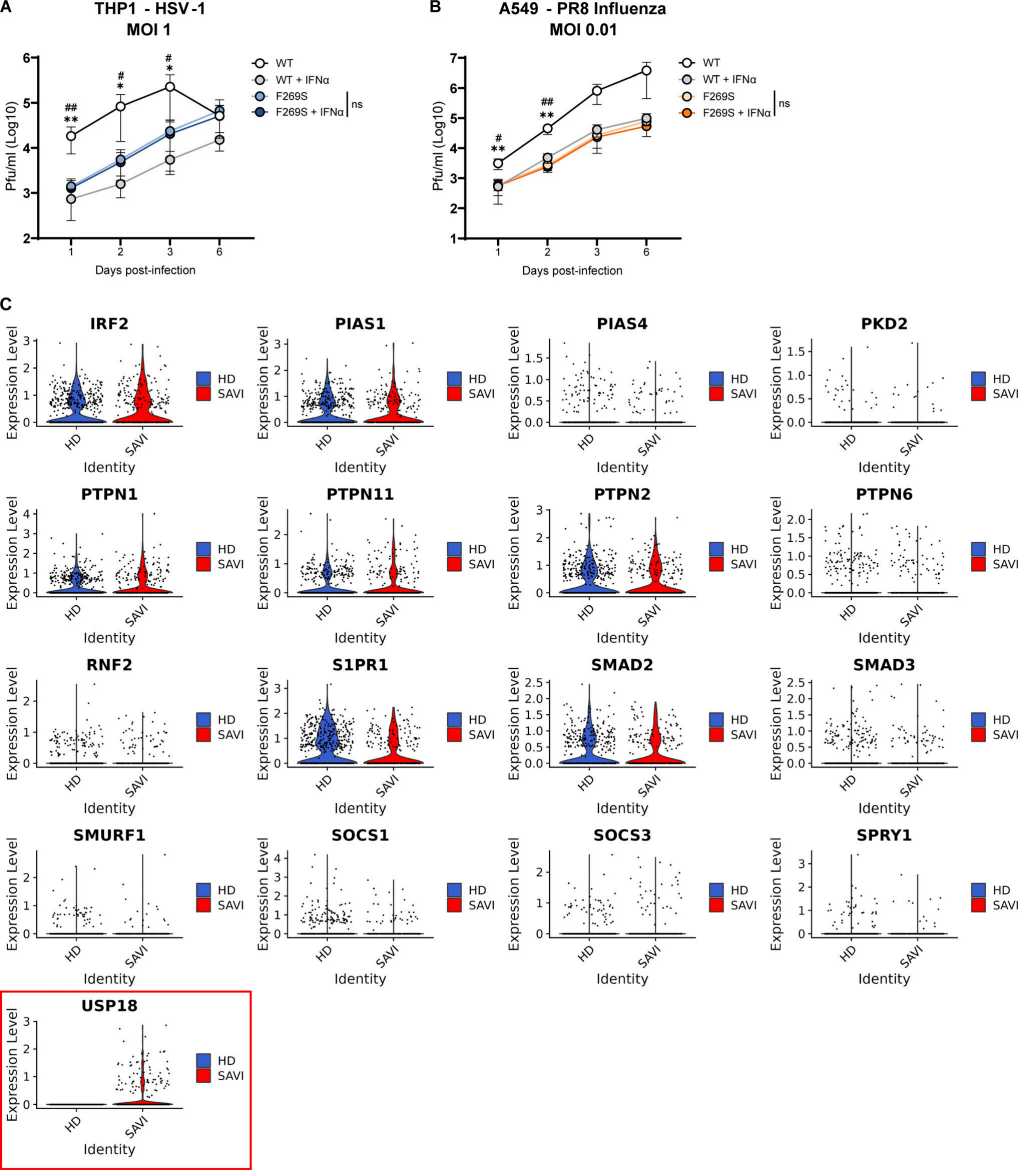
**Negative regulators of type I IFN response in HD and SAVI cells from scRNAseq data. (A and B)** Kinetics of HSV-1 replication in THP1 (A) and IAV replication in A549 (B) infected at the indicated MOI. Cells were pre-stimulated or not with IFNα for 16 h and then inoculated with the indicated infectious viruses. Supernatants were collected at 1, 2, 3, and 6 days after infection and titered on Vero cells (for HSV-1) or MDCK cells (for IAV) (mean ± SD; *n* = 3 independent experiments in technical triplicate for A; *n* = 2 independent experiments in technical triplicate for B; two-way ANOVA with Tukey’s multiple comparisons; * indicates statistical significance between WT and WT+IFNα; *, P < 0.05; **, P < 0.01; ^#^ indicates statistical significance between WT and F269S; ^#^, P < 0.05; ^##^, P < 0.01; ns = not significant). **(C)** Violin plot panels showing the normalized expression level of indicated genes from scRNAseq data in SAVI and HD samples. Violin plot in the red square is also shown in Fig. 7 I.

It has been hypothesized that the enhanced type I IFN signaling observed in SAVI patients may be protective and may explain why in both our patient and the cohort of SAVI patients previously analyzed no opportunistic infections have been recorded (Fremond et al., 2021; Konno et al., 2018). We thus evaluated how the basal immune activation linked to the F269S mutation impacted susceptibility to viral infection. For this purpose, THP1, A549, and HUVEC cells were infected with influenza A virus (IAV), herpes simplex virus type 1 (HSV-1), or Zika virus (ZIKV), respectively, and viral load was evaluated at different time points post-infection. Interestingly, while the viruses strongly replicated in WT cells, STING F269S expressing cells showed basal protection from viral infection (Fig. 7, A–C; and Fig. S5, A and B). Importantly, prior exposure to IFNα strongly reduced infection in WT cells, but no further decrease was observed in STING F269S-expressing cells (Fig. 7, A–C; and Fig. S5, A and B), suggesting that they may be hyporesponsive to innate priming. In agreement, when macrophages derived from the SAVI patient monocytes were challenged with the double-strand RNA analog poly(I:C) or the STING agonist 2′3′-cGAMP, no strong increase in ISG15 expression was observed (Fig. 7 D), similarly to previous reports (Lin et al., 2021). This hyporesponsiveness was confirmed in the different cell line models, where the IFN score did not significantly increase upon poly(I:C) or cGAMP stimulation as compared to STING WT expressing cells (Fig. 7, E–H), suggesting that the impaired response observed in patient cells is not due to the undergoing immunosuppressive treatment, rather it is a cell-intrinsic feature linked to the STING mutation.

To explore the mechanisms behind this hyporesponsiveness, we interrogated our scRNAseq dataset for the expression of a set of previously described negative regulators of type I IFN signaling (Arimoto et al., 2018) in the SAVI patient cells. Most of the genes analyzed resulted in low or similar expression between SAVI and HD control (Fig. S5 C). However, a significant difference in expression was observed for the USP18 gene, which was upregulated only in SAVI patient cells (Fig. 7, I–K; and Fig. S5 C). Interestingly, USP18 upregulation has been shown to contribute to the hyporesponsiveness of cells from Down syndrome patients to a second IFN stimulation (Malle et al., 2022). We thus explored the contribution of USP18 to the hyporesponsiveness in SAVI cells by depleting it in A549 (Fig. 7 L). However, depletion of USP18 did not revert the phenotype as no re-gained response to poly(I:C) was observed (Fig. 7 M), indicating no major role of this negative regulator in SAVI-associated hyporesponsiveness in this experimental model.

Overall, these data show that the basal type I IFN levels observed in SAVI patients confer protection against viral infection, while they are not further increased in response to exogenous triggers. Continuous type I IFN signaling in SAVI cells may activate a negative feedback loop through mechanisms that remain to be elucidated, or the pathway may have reached its maximum activation, ultimately resulting in a decreased response to subsequent stimulation.

## Discussion

STING is a key player in orchestrating type I IFN responses when it senses cytosolic DNA, whether it originates from foreign pathogens or the host itself (Ishikawa and Barber, 2008; Burdette et al., 2011; Yu and Liu, 2021; Abe et al., 2013). In our study, we report the identification of a de novo heterozygous *STING1* variant (F269S) not described in the literature and resulting in a GOF phenotype leading to autoinflammation and the development of SAVI disease. Our findings expand the spectrum of *STING1* mutations associated with SAVI pathogenesis and provide mechanistic insight into how aberrant STING activation can cause disease.

The scRNAseq performed for the first time on mononuclear cells (MNC) from the BM of SAVI patient carrying the novel mutation in STING, prior to initiation of JAK-inhibitor treatments, revealed dysregulated type I IFN and inflammatory pathways in all immune cell populations, including the most primitive HSC compartment, and highlighted a strong cell death response, especially in the T and B cells compartments, resulting in T cell cytopenia and a prevalence of naïve T cells in the circulation.

Overexpression of STING F269S recapitulated the patient cell phenotypes with increased IFN scores and inflammation in both hematopoietic and non-hematopoietic cell types. Mechanistically, we demonstrated through our in vitro models that the substitution of the phenylalanine in position 269 with serine causes spontaneous STING trafficking from the ER to the GOLGI without the requirement of a ligand, resulting in constitutive activation of STING signaling. From the early first reports of STING GOF mutations causing SAVI disease (Jeremiah et al., 2014; Liu et al., 2014), several mutations have been identified spanning exons 5–7, mainly clustering in two regions of the STING protein. Mutations like V147L/M, F153V, N154S, V155M, and G158A localize at the dimerization interface of STING (Liu et al., 2014; Jeremiah et al., 2014; Munoz et al., 2015; Seo et al., 2017; Raffaele et al., 2020; Tang et al., 2020; Staels et al., 2020), while mutations such as C206Y/G, G207E, R281Q/W, and R284G/S localize in the LBD, outside the dimer interface, at the dimer–dimer contact sites observed during STING oligomerization (Saldanha et al., 2018; Lin et al., 2020; Keskitalo et al., 2019). According to the available structural information of STING, it has been proposed that mutations within the dimerization interface could promote the rotation of the LBD resulting in STING oligomerization. Conversely, the other residues may relieve the autoinhibition of STING oligomerization, thereby promoting its activation (Lin et al., 2020; Ergun et al., 2019; Konno et al., 2018; Liu et al., 2023b; Shang et al., 2019). We showed that the de novo mutation falls in this latter category, as F269 localizes in close proximity to R281 and R284 at the interdimer oligomerization interface, suggesting that mutations of this residue may affect the conformation of the inactive STING. Further studies are needed to identify the precise structural consequences of this novel mutation, potentially providing new insight into the mechanism of STING autoinhibition by the C-terminal tail (Ergun et al., 2019).

Although SAVI is considered a type I interferonopathy, the contribution of type I IFN in driving the pathology is not completely understood. Works in SAVI mouse models have shown that immune cell abnormalities, lung inflammation, and fibrosis develop independently from IFNα/β receptor signaling (Motwani et al., 2019; Warner et al., 2017). Additionally, a role for type II IFN receptors in autoinflammation and immune dysregulation mediated by STING activation has been reported in mice (Stinson et al., 2022). Data from SAVI patients support increased levels of ISGs in blood cells; however, the role of type II IFN has not been clearly addressed. We show here that the de novo F269S STING variant is associated with elevated ISG scores and demonstrates a clear dependency of this signature on type I IFN, with no role observed for IFNγ. It is to be noted that while we show that this phenotype seems to be recapitulated in PBMC from the patient, most of the data that support this conclusion are derived from overexpression systems, and experiments in patient cells are limited due to sparse sample availability. Additional experiments across patients and more physiological expression systems will help confirm that type II IFN does not drive elevated IFN scores in humans.

Phenotypic differences have been observed among individuals with STING GOF mutations with no obvious genotype–phenotype correlation. The patient experienced the onset above 1 year of age, despite most commonly the first signs of the disease are already present (Fremond et al., 2021). Moreover, she did not experience either signs of systemic vasculitis or opportunistic infections. The patient presented with DAH due to ANCA-associated vasculitis. Thus far, only two SAVI patients with DAH have been described. The first case involved a patient with the V155M mutation who presented with respiratory manifestations and ILD. DAH was related to pulmonary vasculitis with positive autoantibodies (Tang et al., 2020). More recently, a new case of SAVI presenting massive intra-alveolar hemorrhage was reported in a 4-year-old patient, carrying the R281Q variant, thus diagnosed with SAVI (Ladoux et al., 2023). However, she resulted negative for autoantibodies. Despite being less common among SAVI patients, alveolar hemorrhage is a main feature of COPA syndrome (Watkin et al., 2015), another interferonopathy physio-pathologically related to SAVI, and it has been observed also in systemic lupus erythematosus patients (Esper et al., 2014; Pego-Reigosa et al., 2009). Our additional reported case of DAH in a SAVI patient further strengthens the importance of a correct diagnosis in patients presenting with DAH.

From the immunological point of view, T cell cytopenia is a clinical hallmark of SAVI (Liu et al., 2014; Fremond et al., 2021), which is recapitulated also in the mouse model of the disease. Importantly, this phenomenon occurs independently of type I IFN signaling (Warner et al., 2017; Siedel et al., 2020; Luksch et al., 2019; Motwani et al., 2019; Bouis et al., 2019). In addition, an imbalance between naïve and memory T cells, and proliferation defects have previously been associated with SAVI (Cerboni et al., 2017). In accordance, our patient displayed low T cell counts, and deficiency in memory CD4 and CD8 T cells with prevalent, almost exclusive, naïve T cells in circulation. A partial defect in T cell proliferation was also observed, in line with the intrinsic antiproliferative action of activated STING reported in patient cells and mouse models (Cerboni et al., 2017) and the significant reduction of CD8^+^ memory T cells. Our scRNAseq data further confirmed the reduced proportion of the T cell compartment and highlighted a strong apoptotic signature in both CD4^+^ and CD8^+^ T cells. This observation is consistent with the pro-apoptotic role of STING as described by Gulen et al. (2017) where STING signaling strength promotes apoptosis independently of the IFN pathway. More recently, a novel mechanism linking STING activation with T cell death was described, in which STING GOF mutants induce ER stress and UPR, priming T cells to become hyper-responsive to TCR signaling (Wu et al., 2019). As our transcriptomic data indicated the upregulation of UPR genes in T cells, we further investigated UPR-mediated cell death in SAVI T cell models. UPR and proapoptotic genes were similarly upregulated in both WT and mutant cells upon TCR stimulation; however, a robust increase in cell death was observed only in F269S JTC. This suggests that the STING GOF mutation primes cells for TCR-mediated cell death. Although the detailed mechanisms that sensitize SAVI cells for apoptosis remain to be uncovered, we showed that this phenotype is independent of IFNα/γ and TNFα secretion, as well as from autophagy-dependent cell death mechanisms. High STING signaling–mediated T cell apoptosis has also been recently associated with impaired establishment of T cell memory phenotype upon antigen stimulation (Quaney et al., 2023), supporting the hypothesis that activated STING F269S T cells die before transitioning to an effector state due to hyperactivation and apoptosis. Of note, both apoptosis, UPR genes, and p53-related genes were among the strongly upregulated pathways in the B cell compartment, suggesting that hyperactive STING may also prime B cells to apoptotic cell death. Thus far, works in mouse models have highlighted a central role for T but not B cells in SAVI lung pathology (Gao et al., 2022; Luksch et al., 2019), and no major alterations in the B cell phenotype were observed in our patient, leading us to focus mainly on the cell-intrinsic mechanism of F269S STING activation in T cells. Nevertheless, apoptosis following stimulation with STING agonists has been reported also in primary and malignant B cells (Tang et al., 2016). Thus, it will be of interest to further dissect the impact of STING GOF mutants in this cell compartment.

Lung involvement is a major feature in SAVI pathology with >75% of patients presenting ILD (Fremond and Crow, 2021). Lung fibrosis and respiratory symptoms are frequently reported in SAVI patients with described cases of pulmonary vasculitis (Tang et al., 2020). Mouse models of the disease have been generated to elucidate SAVI pathogenesis. While these models do not fully replicate the phenotype of the human disease, they have highlighted a central role of hematopoietic-derived cells in lung disease, with a more prominent role observed for T cells (Warner et al., 2017; Martin et al., 2019; Gao et al., 2022). Recently, it has been shown that endothelial cells play a crucial role in the initiation of the disease by recruiting immune cells to the lungs (Gao et al., 2022; Gao et al., 2023, *Preprint*). In line with the reported activation phenotype, we show here that expression of the STING F269S variant in primary endothelial cells is sufficient to upregulate IFN response and adhesion molecules, highlighting endothelial inflammation and a possible enhanced immune recruitment through increased adhesion as contributing factors to SAVI. Interestingly, these phenotypes were recapitulated and even enhanced through IFNα- and TNFα-dependent mechanisms when endothelial cells were treated with supernatant from the SAVI promonocytic cell model. While only IFNα contributes to the upregulation of ISGs in endothelial cells, both IFNα and TNFα trigger endothelial inflammation, activation, and damage. Overall, our data suggest that endothelial–immune cell interaction may further boost immune infiltration and contribute to endothelial damage and exacerbation of the lung phenotype. Nevertheless, we recognize the limitation of our models which are mainly based on protein overexpression and believe that future investigation of this crosstalk in relevant disease models will help dissect further key cytokines and mediators that drive the pathology in vivo. Of note, STING F269S expression upregulated ISGs also in epithelial cells, and further work in vitro and in vivo is needed to evaluate the potential contribution of this cell compartment to SAVI pathogenesis.

After diagnosis, the patient started baricitinib with amelioration of the main clinical manifestations. Although we do not have data from the peripheral blood cells of the patient before the initiation of the clinical treatment, we observed a persisting basal higher expression of ISGs during treatment, thus suggesting an incomplete effect of baricitinib in controlling the IFN signature in this patient, despite good clinical response. In agreement, while Jak inhibitors such as ruxolitinib, baricitinib (JAK1/2 inhibitors), and tofacitinib (JAK1/3 inhibitor) have been demonstrated as valid therapeutic approaches for the treatment of the multisystemic inflammation associated with STING GOF, no significant modulation of the IFN signature was reported in most of the patients, with only partial or absent decrease in the IFN scores (Volpi et al., 2019; Fremond et al., 2016; Sanchez et al., 2018; Wan et al., 2022). These data may indicate that IFNα is not the unique or main driver of pathological manifestations. Indeed, SAVI pathology can develop independently of IFNα signaling through IFNγ in mouse models of the disease (Stinson et al., 2022). However, differences between humans and mice may exist, as we observed no impact of IFNγ on either the immunological or endothelial phenotypes in the context of STING over-expressing models as well as patient-derived PBMC. In line with the concept that elevated IFN scores may not drive pathology in SAVI, we have observed that anti-inflammatory treatments curtailing neurotoxicity in models of another type I interferonopathy, the AGS, do not lower the typical ISG scores in cells of the human CNS (Giordano et al., 2022). In agreement, we identified here TNFα as a mediator of pathological inflammation in endothelial cells upon STING activation. Although TNFα is not directly involved in JAK/STAT signaling, there is evidence of indirect contributions of this cytokine to the pathway (Hu et al., 2021) that may explain why SAVI patients tend to benefit from JAK inhibitor treatment despite sustained IFN scores. The identification of these cytokines in the context of overexpression systems will require further investigation as physiological levels of the mutated STING may contribute differently to the phenotype in vivo. Overall, our data suggest that cytokines, other than IFNs, may contribute to the SAVI phenotypes and yet-to-be-identified proinflammatory mediators could further contribute to this phenotype in the more complex in vivo setting. Interestingly, recent findings indicate that STING functions also as a proton channel that regulates the Golgi pH (Liu et al., 2023a). Given that proton leakage from organelles is a common signal for non-canonical inflammasome activation, increased STING activity at the Golgi could therefore contribute to fueling inflammatory cascades through altered proton homeostasis also in SAVI cells.

Patients treated with JAK inhibitors experience episodes of respiratory infections (Sanchez et al., 2018; Volpi et al., 2019), suggesting an increased susceptibility to viral infections and supporting the need for safer and more efficient treatments. Inhibitors like H151, which block STING palmitoylation ameliorate multiorgan inflammation in AGS, psoriasis, and other disease models (Haag et al., 2018; Yu et al., 2020; Hansen et al., 2019). TBK1 antagonists represent another therapeutic option for the treatment of autoimmune diseases and interferonopathies (Hasan and Yan, 2016). We show here that both H151 and TBK1 inhibitors efficiently downregulate ISG expression in SAVI cells, with a variable efficacy from 60 to 100% reduction, suggesting that these inhibitors may represent an alternative strategy for controlling disease manifestations and progression, acting upstream to the IFNAR and JAK/STAT pathway that remain thus functional. Further tests in patient blood cells and appropriate dose and timing will be required to assess their efficacy and safety.

Interestingly, the expression of STING F269S seemed to confer a cell-intrinsic protection to viral entry and spread. It is likely that other STING GOF behave similarly, as only rare cases of infections have been recorded in SAVI patients thus far (Fremond et al., 2021). Further work with patient cells from different genetic backgrounds may shed light on the mechanisms behind this enhanced resistance, likely related to higher basal ISG levels that create an innate barrier to infections. Nevertheless, we also show here that both patient cells and SAVI cell models have an impaired response to exogenous immune stimulation. We hypothesized that this could be due to a negative feedback regulation of the type I IFN pathway related to the specific upregulation of USP18 in SAVI cells. In line, USP18 has been suggested to participate in similar mechanisms of inhibition of type I IFN priming in the context of Down syndrome patients (Malle et al., 2022). However, we could not observe any regained responsiveness upon depletion of USP18 in SAVI A549 cells, suggesting that either other factors may be involved in controlling excessive activation of the pathway or that the pathway in the SAVI lines has already reached the maximum activation capacity and cannot be further enhanced by exogenous triggers.

Finally, our study uncovers for the first time aberrant type I IFN and inflammatory signatures also in the primitive HSC compartment of SAVI patients. Chronic exposure of HSC to inflammatory cues such as IFNα, poly(I:C), or TNFα has been shown to lead to increased proliferation and myeloid skewing at the expense of self-renewal ad repopulating capacity in several preclinical models (Essers et al., 2009; Lopez et al., 2022; Dybedal et al., 2001) With the advancement of therapeutic strategies that limit pathology but not necessarily activation of type I IFN responses, it will be of interest to evaluate the potential long-term consequences of chronic IFN activation on the HSC and progeny over time as the life expectancy of SAVI patients improves. Overall, we describe and mechanistically characterize a new STING mutant that causes SAVI disease providing insight into how aberrant STING activation can cause pathology through the involvement of the myeloid and endothelial compartments. Our results also further the understanding of lack of correct T cell maturation in SAVI patients, provide evidence that constitutive type I IFN signaling leads to a paradoxical impairment of innate immune priming during infection, and, for the first time describe aberrant IFN signatures in the HSC compartment that may impact their long-term functionality. Together, these results will inform the development of improved therapeutic strategies for SAVI and potentially also other type I interferonopathies.

## Materials and methods

### Ethical approval

The studies involving human participants were conducted in accordance with the principles expressed in the Helsinki Declaration, reviewed, and approved by San Raffaele Hospital Ethical Committee. The participants or their parents provided written informed consent to participate in this study (Protocol TIGET09 and TIGET06). Written informed consent was obtained from all the individuals for the publication of any data included in this article.

### Primary cells

Human primary MNC were isolated from total blood collected upon informed consent by Ficoll gradient. Total PBMC were maintained in IMDM medium (Gibco) supplemented with penicillin (100 U/ml), streptomycin (100 mg/ml), 2% glutamine, 10% fetal bovine serum (FBS), MEM Non-Essential Amino Acids Solution (1×) (11140035; Gibco), and sodium pyruvate (1×) (11360039; Gibco). CD14^+^ monocytes were isolated through positive magnetic bead selection according to the manufacturer’s instructions (CD14 MicroBeads, human; 130050201; Miltenyi). MDM were differentiated from isolated CD14^+^ cells in DMEM supplemented with 10% FBS, penicillin (100 IU/ml), streptomycin (100 mg/ml), 2% glutamine, and 5% human serum AB (35-060-CI; Corning) for 7 days. For T cell culture, PBMC depleted of the CD14^+^ fraction were activated using magnetic beads conjugated to anti-human CD3 and CD28 antibodies (Dynabeads Human T-Expander CD3/CD28; 11141D; Gibco) in IMDM medium supplemented with penicillin (100 IU/ml), streptomycin (100 mg/ml), 2% glutamine, 10% FBS, and 5 ng/ml of IL-7 (167200-07-B; PeproTech) and IL-15 (167200-15-B; PeproTech) for 3 days and then left without beads stimulation. For scRNAseq analysis, MNC were isolated by density gradient centrifugation from BM samples collected upon informed consent from healthy volunteers and immediately processed for subsequent analysis as detailed in the scRNAseq method section.

### Cell lines

THP-1 and JTC were maintained in RPMI medium (Corning) supplemented with 10% FBS, penicillin (100 IU/ml), streptomycin (100 μg/ml), and 2% glutamine. A549 cells were maintained in DMEM 1X (Sigma-Aldrich) supplemented with 10% FBS, penicillin (100 IU/ml), and streptomycin (100 μg/ml). HUVEC were maintained in Medium M199 1× + GlutaMA-I 38 (Gibco) supplemented with 20% FBS, 1% penicillin (100 IU/ml), streptomycin (100 μg/ml), 1% glutamine, 1% HEPES (15630080; Thermo Fisher Scientific), 1% Endothelial Cell Growth Supplement (02-102; Sigma-Aldrich) 0.2µ-filtered, and 0.1% heparin (H3149; Sigma-Aldrich).

All cells were maintained in a 5% CO_2_ humidified atmosphere at 37°C.

### Proliferative response assay

PBMC isolated by Ficoll gradient, were cultured in X-VIVO 15 (LONZA) supplemented with 5% human serum (EuroClone S.p.A.) alone or in the presence of polyclonal stimuli (anti CD3 moAb ± anti CD28 moAb; IL2 in suboptimal dose; PHA or PWM). After 72 h, a solution containing tritiated thymidine (3H-Thy) was added and the culture was prolonged for an additional 16–18 h incubation. The amount of 3H-Thy incorporated by neosynthesized DNA, proportional to cell proliferation, was measured by a BetaCounter (MicroBeta Trilux, Perkin Elmer Life Sciences) and expressed in counts per minute (c.p.m.). The reported stimulation index (S.I.) was calculated as the ratio between c.p.m. of stimulated cells and c.p.m. of cells proliferating spontaneously in the absence of stimuli.

### Plasmids

The human STING WT and F269S mutant STING sequences were amplified from HD and patient cDNA by PCR using primers with homologies for the STING sequence and carrying NheI and BamHI restriction site sequences (Fw primer: 5′-ATT​AGC​TAG​CGC​CAC​CAT​GCC​CCA​CTC​CAG​CCT​GCA-3′; Rev primer: 5′-ATT​AGG​ATC​CTT​ATC​AAG​AGA​AAT​CCG​TGC​GGA​GAG​GGA​GGG​GC-3′). The synthesized sequence was then digested with NheI and BamHI and cloned into the transfer plasmid pCCL.sin.cPPT.SK-T6.Cas9-PGK.Puro.TaV2A.rTTAm3.Wpre after removal of the Cas9 sequence through digestion with the same restriction enzymes. In the resulting plasmid, the STING gene is under the control of a human synthetic promoter regulated by the TetO7 operon, allowing induction of gene expression only in the presence of dox. In addition, the phosphoglycerate kinase (PGK) promoter drives the expression of a puromycin-selectable marker.

### Generation of inducible cell lines

To generate cell lines overexpressing STING WT or STING F269S in an inducible manner, cells were transduced with vectors produced using the above-described transfer plasmids. LV were produced and titered as previously described (Dull et al., 1998; Follenzi and Naldini, 2002). Briefly, LV were produced using the packaging plasmid pMDLg/pRRE, Rev-expressing plasmid pCMV-Rev, the pCCL.sin.cPPT.SK-T6.STING-PGK.Puro.TaV2A.rTTAm3.Wpre or pCCL.sin.cPPT.SK-T6.STINGF269S-PGK.Puro.TaV2A.rTTAm3.Wpre transfer plasmids and the pMD2.VSV-g envelope-encoding plasmid. Cells were transduced at a limiting multiplicity of infection (MOI), calculated by titration of vector stocks on 293T cells and expressed as transducing units/293T cell, to have one vector copy per cell after 0.5–2 µg/µl puromycin selection (P8833; Sigma-Aldrich). STING expression was transiently induced with 1 µg/ml dox administration (D9891; Sigma-Aldrich) for 6 h before washing in THP1 and A549 cells and for 24 h in JTC and HUVEC. Pellets for western blot validation were collected at 6, 12, and 24 h after dox administration.

### Gene expression

RNA extraction from cells was performed using the RNeasy Plus micro-Kit (74034; QIAGEN) or ReliaPrep RNA Cell Miniprep System (Z6011; Promega) according to the manufacturer’s instructions. The extracted mRNAs were reverse transcribed (RT) using the SuperScript Vilo kit (11754-250; Invitrogen). RT-qPCR analyses were performed using TaqMan probes from Applied Biosystems or using probe-free SYBR amplification. PCR was run for 40 cycles using the Viia 7 instrument while the Viia 7 software was then used to extract the raw data (Ct). To determine gene expression, the difference (ΔCt) between the threshold cycle (Ct) of each target gene and that of the reference gene was calculated by applying an equal threshold. Relative quantification values were calculated as the fold-change (FC) expression of the gene of interest in the test sample over its expression in the reference sample by the formula 2^–ΔΔCt^. The expression was normalized using the housekeeping genes HPRT1 or GAPDH. Refer to Table S1 for Taqman probes and Table S2 for SYBR primers sequences used. qPCR-based IFN and inflammation scores were calculated from a panel of five/six ISGs (ISG15, RSAD2, OAS1, USP18, IFI27, IFI44L, and IFIT1) and six inflammation-related genes (IRGs) (CARD8, IL6, TNF, CXCL8, CXCL10, and IL1B). Scores were calculated as the median FC of all genes contributing to the score. In experiments with patient cells, the HD was used as a control sample resulting in an IFN score = 1. In experiments with cell lines, the −dox condition was used as control sample.

### Flow cytometry

All cytometric analyses were performed using the FACS Canto II instrument and analyzed with the FACS Express software (De Novo Software).

### Cell subpopulation composition

To measure CD3^+^ T cells subpopulation composition cells were harvested after 5 days in culture, washed, and resuspended in PBS containing 2% FBS. Cells were then incubated with anti-human receptor blocking antibodies (RRID:AB_2892112) for 10 min at room temperature and then stained for 20 min at 4°C with the following antibodies (anti-CD3: RRID:AB_314060; anti-CD4: RRID:AB_397037; anti-CD8: RRID:AB_1645736; and anti-CD45RA: RRID:AB_2733817; RRID:AB_830800). To exclude dead cells from the analysis, 10 ng/ml 7-aminoactinomycin D (7-AAD) (A9400; Merck) was added to each sample.

### Apoptosis

For cell death experiments, JTC were treated with dox for 24 h, washed, and then stimulated with 10 nM PMA (P8139; Sigma-Aldrich) plus 0.1 µM ionomycin (I9657; Sigma-Aldrich) for an additional 24 h with or without treatment with the indicated inhibitors. Refer to Table S4 for drugs and concentrations used. The apoptosis assay was performed with the Annexin V Apoptosis Detection Kit I (559763; BD) according to the manufacturer’s instructions.

### Western blot

For western blot analysis cells were collected and lysed with radioimmunoprecipitation assay lysis buffer (50 mm Tris-HCl, pH 7.5, 150 mm NaCl, 1% Nonidet P-40, 0.5% sodium deoxycholate; 0.1% SDS) containing 1× protease inhibitor cocktail (11873580001; Sigma-Aldrich) and 1× phosphatase inhibitor (PHOSSTOP; 04906837001; Roche). Total protein concentration in the cell lysates was quantified with Bio-Rad Protein Assay Dye Reagent (5000006EDU; Bio-Rad). Bovine serum albumin (BSA) (A9647; Sigma-Aldrich) was used for the generation of a standard curve. Equal amounts of protein in samples were separated on 4–12% Bis-Tris Plus gels (NW04120BOX; Thermo Fisher Scientific) and transferred to polyvinylidene difluoride membrane (IB401001; Life Technologies) by electroblotting. The nonspecific antibody-binding sites were blocked with 5% BSA in TRIS-buffered saline (TBS) 1× + 0.1% Tween 20 (P1379; Sigma-Aldrich) (TTBS). Blots were then incubated with primary antibody diluted in 5% BSA or 1% BSA for p-STAT1 overnight at 4°C, washed three times with TTBS 1×, and incubated with secondary antibody diluted in 5% BSA. β-Actin was used as a housekeeping protein control. Revelation was done using Immobilon Forte Western HRP Substrate (WBLUF0500; Millipore) according to the manufacturer’s instructions. Refer to Table S3 for the list of antibodies and dilutions. In western blot quantifications, the signal was quantified by densitometry using ImageJ software and normalized to β-actin or histone H3.

### Molecular modeling

Comparative modeling of human STING WT and F269S mutant was carried out using ColabFold (Mirdita et al., 2022). The Phe269S variant was mapped on STING cryo-EM structures downloaded from the Protein Data Bank under accession codes 6NT5 (human apo STING), 6NT8 (chick STING in cGAMP-bound state) (Shang et al., 2019), and 8IK0 (chick STING oligomers) (Liu et al., 2023b). Superpositions were carried out using the SUPERPOSE command in COOT (Emsley et al., 2010). Structural figures were rendered using PyMOL (The PyMOL Molecular Graphics System, Version 2.0 Schrödinger, LLC).

### Immunofluorescence (IF)

Cells were fixed with 4% paraformaldehyde (15670799; Thermo Fisher Scientific) for 20 min at room temperature. To evaluate the colocalization of STING with the Golgi marker GM130, THP1 and JTC cells were permeabilized with 0.1% Triton X-100 (X100; Sigma-Aldrich) for 20 min at room temperature. For blocking nonspecific sites, cells were incubated for 30 min in PBS + 10% FBS and stained overnight at 4°C with primary antibodies. After three washes with 1× PBS, cells were incubated with donkey anti-rabbit IgG, Alexa Fluor 488 or donkey anti-mouse IgG, Alexa Fluor 555 for 1 h at room temperature. Nuclei were stained with DAPI (10236276001; Roche) for 5 min at room temperature. To evaluate γH2AX or cleaved caspase 3 (cC3) staining, HUVEC cells were permeabilized and blocked with PBS-1%BSA-0.3%Triton for 20 min, followed by overnight incubation at 4°C with primary antibodies. After three washes with 1× PBS, cells were incubated with donkey anti-rabbit IgG, Alexa Fluor 555, or donkey anti-mouse IgG, Alexa Fluor 488 for 1 h at room temperature. Nuclei were stained with DAPI for 10 min at room temperature. Refer to Table S3 for the list of antibodies and dilutions.

Images were recorded using the TCS SP5 Leica confocal microscope, 40× with oil, or the Olympus FluoVIEW 3000 RS confocal microscope, 60× with oil. Image analysis was performed using ImageJ.

### ELISA assay

The amount of IP-10 was measured by ELISA (DIP100; Bio-Techne) according to the manufacturer’s instructions in the supernatant of THP1 cells 24 h after induction of STING WT or STING F269S. The absorbance of each sample was determined on a spectrophotometer using a Multiskan GO microplate reader (Thermo Fisher Scientific) and normalized to antigen standard curves.

### Meso scale discovery (MSD) assay

The analysis of the secretome of THP1, JTC, A549, and HUVEC cells was performed by an MSD custom assay in collaboration with the Human Immune Monitoring Shared Resource, University of Colorado, Anschutz Medical Campus. Samples were run at twofold dilution and the analytes were chosen in a U-Plex customizable format: IL-1RA, IL-1β, IL-6, IL-8, IP-10, MCP-1, and TNFα.

### Compounds

All the compounds were resuspended and stored following the manufacturer’s instructions. Refer to Table S4 for the list of drugs and concentrations. For experiments in patient PBMC, cells were treated with αIFNAR or αIFNγ for 24 h immediately after isolation and then collected for gene expression analysis. For stimulation of human MDM, cells were stimulated with indicated stimuli at day 7 of in vitro differentiation for 24 h and then collected for gene expression analysis. For experiments with inhibitors, THP1 cells were treated with dox and the inhibitors for 6 h. After the wash, the inhibitors were re-added at the same concentration, and cells were collected after 24 h from dox induction for gene expression analysis. HUVEC were treated with dox and the inhibitors for 24 h and then collected for gene expression analysis. For stimulation experiments, THP1 and A549 were treated with dox for 6 h, washed, and the different stimuli were added to the medium. After 24 h from dox induction, cells were collected for gene expression analysis. JTC and HUVEC were treated with dox, the different stimuli were added after 6 h, and cells were then collected after 24 h for gene expression analysis.

### CM experiments

THP1 cells were treated with dox for 6 h, washed, and collected after 24 h. The cell suspension was centrifuged at 1,200 rpm for 8 min to remove cellular debris and the supernatant was collected and stored at −80°C until following experiments. After thawing, the conditioned medium was recentrifuged at 1,200 rpm for 8 min prior to addition to the cells. To evaluate the impact of THP1-derived conditioned medium, HUVEC cells were seeded at 2.5 × 10^4^ cells/well in a 24-well plate and treated with a ratio of 40% of conditioned medium from THP1 and 60% of HUVEC culture medium for 24 h before subsequent analysis, in the presence or not of the indicated inhibitors. A medium made of 40% RPMI and 60% M199 was used as the control medium. For DNase I treatment experiments, CM were treated with 2 U DNase I (AM2222; Invitrogen) × 100 μl of medium along with 10× DNase I Buffer and incubated for 30 min at 37°C prior to the addition to HUVEC cells.

### Scratch assay

HUVEC cells were seeded at 2.5 × 10^4^ cells/well in a 24-well plate until confluency. Cells were treated with control or conditioned medium for 24 h and then with 10 uM Antimycin A (A8674; Sigma-Aldrich) for 1 h to block proliferation. A line scratch wound was performed with a sterile tip of a micropipette in each well. Cell debris was removed with a PBS wash. Bright-field images were captured at 3, 6, 12, and 24 h. The wound area was quantified by ImageJ software and the percentage of wound closure was calculated by the formula of (W_0_ − W_t_)/W_0_, where W_0_ represents the initial wound area and W_t_ represents the wound area at different time points.

### Knock-down experiment

For the knock-down of USP18 in A549 F269S cells the following reagents were used: ON-TARGETplus human USP18 (11274) siRNA (FE5L013239020005; Dharmacon), ON-TARGETplus non-targeting pool (FE5D0018101005; Dharmacon), and DharmaFECT 1 siRNA transfection reagent (FE5T200103; Dharmacon). 4 × 10^4^ A549 cells/well were seeded in a 48-well plate. The day after, the medium was replaced with fresh complete DMEM medium and the transfection mix was prepared. siRNA and DharmaFECT transfection reagent were diluted, mixed, and added to the cells following the manufacturer’s instructions. siRNAs were used at 50 nM final concentration and 1 µl of transfection reagent/reaction was used. After transfection, cells were incubated at 37°C for 48 h prior to western blot analysis. At 48 h cells were treated with dox with or without poly(I:C) for 24 h and then collected for gene expression analysis.

### Viral infections

#### THP1 infection with the recombinant HSV-1 KCZ virus

THP1 cells were seeded at a density of 1 × 10^5^ cells per well in 48-well plates, each well containing 150 µl of RPMI (complete medium) supplemented with 1% FBS. After seeding, the recombinant HSV-1 KCZ virus (Pinna et al., 2008) was added at a MOI of 1 in a final volume of 100 µl. After the virus was allowed to be adsorbed for 1 h, 400 µl of RPMI supplemented with 2% FBS were added. At different time points after infection, cell culture supernatants were collected and stored at −80°C until the determination of viral titers using a plaque-forming assay in Vero cells.

#### HUVEC infection with ZIKV

HUVEC cells were plated at 2.5 × 10^4^ cells per well in 24-well plates in DMEM (complete medium) supplemented with 2% FBS. 24 h later, ZIKV PRVABC59 strain (GenBank accession #KU501215) was added at a MOI of 0.1 in a final volume of 200 µl of medium supplemented with 2% FBS. After 2 h of infection, the inoculum was removed and a volume of 500 µl of complete medium was added. Cell culture supernatants were collected at 72 h after infection and stored at −80°C until the determination of viral titers using a plaque-forming assay in Vero cells.

#### A549 infection with IAV

A549 cells were plated at 7.5 × 10^4^ cells per well in 24-well plates in DMEM (complete medium) without FBS. 24 h later, the IAV A/PR/8/1934 (H1N1) strain was added at a MOI of 0.01 in a final volume of 200 µl of medium without FBS. After 1 h infection, the inoculum was removed, cells were washed, and a volume of 500 µl of medium without FBS was added. Cell culture supernatants were collected at different time points after infection and stored at −80°C until the determination of viral titers using a plaque-forming assay in MDCK cells.

#### Plaque-forming assays in Vero and MDCK cells

Plaque-forming assays were conducted in Vero cells for HSV-1 and ZIKV and in MDCK cells for IAV, as previously detailed (Pagani et al., 2018, 2022; Pinna et al., 2008). In brief, confluent cells were exposed to serial dilutions of viral stock. Subsequent to incubation, the viral inoculum was aspirated, and in the case of Vero cells, methylcellulose was overlaid in the plaque assay, while MDCK cells were overlaid with a mixture of MEM 2X–Avicel (FMC Biopolymer) supplemented with TPCK-treated trypsin (1 µg/ml). Following the incubation period, cells were stained, and plaques were counted using a stereoscopic microscope (SMZ-1500; Nikon Instruments). The viral titer was calculated in terms of PFU/ml.

### Generation and analysis of scRNAseq

#### Data generation

scRNAseq libraries were generated using a microfluidics-based approach on Chromium Controller (10x Genomics) using the Chromium Single Cell 3′ Reagent Kit v3.1 according to the manufacturer’s instructions. Briefly, single cells from BM-derived MNC were suspended in 0.4% BSA-PBS at a concentration ranging from 800 to 1,200 cells/μl. 8,000 cells were added to each channel to achieve a recovery rate of 5,000 cells per sample. Cells were partitioned in Gel Beads in Emulsion and lysed, followed by RNA barcoding, RT, and PCR amplification (10 cycles). The concentration of the scRNAseq libraries was determined using Qubit v3.0, and size distribution was assessed using an Agilent 4200 TapeStation system. Libraries were sequenced on an Illumina NovaSeq instrument (paired-end, 150-bp read length).

#### Data processing and graph-based clustering

Raw data from scRNAseq was analyzed and processed into a transcript count matrix by Cell Ranger (https://support.10xgenomics.com/single-cell-gene-expression/software/pipelines/latest/what-is-cell-ranger, v4.0.0) from the Chromium Single Cell Software Suite by 10x Genomics. Fastq files were generated using the Cell Ranger “mkfastq” command with default parameters. Gene counts for each cell were quantified with the Cell Ranger “count” command with default parameters. For all analyses, the human genome (GRCh38.p13) was used as the reference. The resultant gene expression matrix was imported into the R statistical environment for further analysis. Cell filtering, data normalization, and clustering were carried out using the R package Seurat v3.2.2 (Stuart et al., 2019). For each cell, we calculated the following quality measures: the percentage of mitochondrial genes, the total read count in genes, and the number of expressed genes. Cells with a ratio of mitochondrial versus endogenous gene expression >0.2 were excluded as putative dying cells. Cells expressing <200 or >6,000 total genes were also discarded as putative poorly informative cells and multiplets. Counts were normalized using Seurat function “NormalizeData” with default parameters. Expression data were then scaled using the “ScaleData” function, regressing on the number of the unique molecular identifier, percentage of mitochondrial gene expression, and difference between S and G2M scores. Cell cycle scores were calculated using the “CellCycleScoring” function. The different single-cell datasets were integrated into a single object using the R package Harmony v1.0 (Korsunsky et al., 2019) for dealing with experimental and biological confounding factors and removing batch effects. Dimensionality reduction was then performed with principal component analysis on the batch-corrected data. Uniform Manifold Approximation and Projection (UMAP) dimensionality reduction (Mcinnes et al., 2018) was performed on the calculated principal components to obtain a 2D representation for data visualization. Cell clusters were identified using the Louvain algorithm at resolution r = 1.2, implemented by the “FindCluster” function of Seurat. Clusters annotation was identified using the R/Bioconductor package SingleR v1.4.0 (Aran et al., 2019) against the BlueprintEncodeData dataset (a combined human bulk RNA-seq dataset from Blueprint and ENCODE) and manually verified by querying known markers.

#### Differential expression and GSEA

To find the differentially expressed (marker) genes for the annotated clusters, the functions “FindAllMarkers” (iteratively comparing one cluster against all the others) and “FindMarkers” (two condition comparison) from the Seurat package were used with default parameters. Significant differentially expressed genes were identified using the following parameters: adjusted P values <0.05, average logFC > 0.25, and percentage of cells with expression >0.1. Downstream analysis, including GSEA, was performed with R/Bioconductor package ClusterProfiler using a list of databases including Gene Ontology, Kyoto Encyclopedia of Genes and Genomes Pathway Database, Reactome Pathway Database, and Molecular Signatures Database. Enriched terms with a q value <0.05 were considered statistically significant. Heatmaps were produced using the R package pheatmap. Heatmaps were produced using the R/Bioconductor package ComplexHeatmap (Gu et al., 2016). Charts and images were produced using R package ggplot2.

#### IFN score calculation from scRNAseq

IFN scores derived from the single-cell sequencing expression data were calculated in selected 28 ISGs using the z-score-based standardized IFN score calculation method as reported in (Tüngler et al., 2016; Rice et al., 2013; Lambers et al., 2019; Kim et al., 2018). In detail, Z-scores for each of the 28 genes were calculated between the SAVI and HD samples in the different annotated cell types using the following equation:

$$Z-score\;for\;each\;gene=\frac{\left[gene\;count-mean(HD\;gene\;expression)\right]}{SC(HD\;gene\;expression)}.$$

As the calculation for each z-score is relative to the mean and standard deviation of the HD sample, each z-score can become negative if the gene expression is below the mean of that in the HD sample.

### Statistics

All statistical analyses were conducted with GraphPad Prism 9 version. In all studies, values are expressed as mean ± standard error of the mean (SEM) or mean ± standard deviation (SD) and all *n* numbers represent biological repeats. Statistical analyses were performed as indicated in the figure legends. Differences were considered statistically significant at *P < 0.05, **P < 0.01, ***P < 0.001, ****P < 0.0001, “ns” represents non-significance.

### Online supplemental material

Fig. S1 shows additional analysis from scRNAseq of SAVI patient cells. Fig. S2 shows the characterization of the SAVI cell lines generated in this study. Fig. S3 shows additional cell death experiments performed in the SAVI JTC. Fig. S4 shows additional experiments performed in HUVEC cells treated with CM with or without inhibitors. Fig. S5 shows the kinetics of viral infection in the SAVI cell lines and violin plots for the expression of negative regulators of type I IFN response from scRNAseq data. Table S1 contains all the Taqman probes used in this work. Table S2 shows all the sequences of the PCR primers used in this work. Table S3 lists all the western blot and IF antibodies used. Table S4 shows information about the drugs used.

## Supplementary Material

Table S1 lists taqman probes.

Table S2 lists sequences of PCR primers.

Table S3 lists antibodies.

Table S4 lists drugs.

SourceData F3 contains original blots for Fig. 3.

SourceData F7 contains original blots for Fig. 7.

SourceData FS2 contains original blots for Fig. S2.

SourceData FS3 contains original blots for Fig. S3.

## Data Availability

The raw and processed data from the scRNAseq experiment have been deposited to the Gene Expression Omnibus (GEO) archive under the accession code GSE267837. Any additional information required to reanalyze the data reported in this article is available from the lead contact upon request.
