# Supplementary material for: A novel STING variant triggers endothelial toxicity and SAVI disease
Source: J Exp Med. 2024 Jul 2;221(9):e20232167. doi: 10.1084/jem.20232167 (PMC11217899; doi:10.1084/jem.20232167)
Supplement: Table S1 — lists taqman probes. [file JEM_20232167_TableS1.docx]

Table S1. List of taqman probes

| **Probe** | **Company** | **Code** |
| --- | --- | --- |
| GAPDH | Thermo-Fisher | Hs02786624_g1 |
| HPRT1 | Thermo-Fisher | Hs01003267_m1 |
| CXCL8 | Thermo-Fisher | Hs00174103_m1 |
| IFIT1 | Thermo-Fisher | Hs01675197_m1 |
| IL1 beta | Thermo-Fisher | Hs01555410_m1 |
| ISG15 | Thermo-Fisher | Hs01921425_s1 |
| CARD8 | Thermo-Fisher | Hs01088221_m1 |
| CASP1 | Thermo-Fisher | Hs00354836_m1 |
| RSAD2 | Thermo-Fisher | Hs00369813_m1 |
| IFI27 | Thermo-Fisher | Hs01086373_g1 |
| IL-6 | Thermo-Fisher | Hs00174131_m1 |
| TNFa | Thermo-Fisher | Hs00174128_m1 |
| CXCL10 | Thermo-Fisher | Hs00171042_m1 |
| IFI44L | Thermo-Fisher | Hs00915292_m1 |
| VCAM-I | Thermo-Fisher | Hs01003372_m1 |
| ICAM-I | Thermo-Fisher | Hs00164932_m1 |
| E-SELECTIN | Thermo-Fisher | Hs00174057_m1 |
| CXCL9 | Thermo-Fisher | Hs00171065_m1 |
| CXCL11 | Thermo-Fisher | Hs00171138_m1 |
