# Supplementary material for: A novel STING variant triggers endothelial toxicity and SAVI disease
Source: J Exp Med. 2024 Jul 2;221(9):e20232167. doi: 10.1084/jem.20232167 (PMC11217899; doi:10.1084/jem.20232167)
Supplement: Table S2 — lists sequences of PCR primers. [file JEM_20232167_TableS2.docx]

**Table S2. Sequences of PCR primers**

| **Gene** | **Sequence (5’ 3’)** |
| --- | --- |
| GAPDH | Forward: CCACCCATGGCAAAT TCC  Reverse: TGGGAT TTCCAT TGATGACAAG |
| HPRT1 | Forward: TGACACTGGCAAAACAATGCA  Reverse: GGTCCTTTTCACCAGCAAGCT |
| IFI27 | Forward: TGGCCAGGATTGCTACAGTTG  Reverse: TATGGAGGACGAGGCGATTC |
| IFI44L | Forward: GCACAGTCCTGCTCCTTCTGCC  Reverse: GGTGGGTCCAGTTGGGTCTGGA |
| IFIT1 | Forward: GGGCATCCTCATGACCATTGGA  Reverse: GGCTACTAGTAACCCCGTTTTTCCTG |
| IFITM1 | Forward: GGGCATCCTCATGACCATTGGA  Reverse: GGCTACTAGTAACCCCGTTTTTCCTG |
| IL1β | Forward: CCACAGACCTTCCAGGAGAATG  Reverse: GTGCAGTTCAGTGATCGTACAGG |
| IL8 | Forward: ACTGAGAGTGATTGAGAGTGGAC  Reverse: AACCCTCTGCACCCAGTTTTC |
| ISG15 | Forward: GAGAGGCAGCGAACTCATCT  Reverse: CTTCAGCTCTGACACCGACA |
| OAS1 | Forward: GGACTGAGGAAGACAACCAGGT  Reverse: AGGAAAGGTGCTTCCGAGGTAG |
| P21 | Forward: GCAGACCAGCATGACAGATTT  Reverse: GGATTAGGGCTTCCTCTTGGA |
| RSAD2 | Forward: CGTGAGCATCGTGAGCAATG  Reverse: TCCCTACACCACCTCCTCAG |
| STING | Forward: CCTGAGTCTCAGAACAACTGCC  Reverse: GGTCTTCAAGCTGCCCACAGTA |
| TNF | Forward: AGGCGGTGCTTGTTCCTCAG  Reverse: GGCTACAGGCTTGTCACTCG |
| BAX | Forward: TGGCAGCTGACATGTTTTCTGAC  Reverse: TCACCCAACCACCCTGGTCTT |
| BAK | Forward: ACGCTATGACTCAGAGTTCC  Reverse: CTTCGTACCACAAACTGGCC |
| CHOP | Forward: GAACCAGGAAACGGAAACAG  Reverse: ACCATTCGGTCAATCAGAGC |
| GADD34 | Forward: ACCTCTACTTCTGCCTTGTCTCC  Reverse: TGGCTCCTTTACTTCTTTCTGTT |
