# Supplementary material for: A novel STING variant triggers endothelial toxicity and SAVI disease
Source: J Exp Med. 2024 Jul 2;221(9):e20232167. doi: 10.1084/jem.20232167 (PMC11217899; doi:10.1084/jem.20232167)
Supplement: Table S3 — lists antibodies. [file JEM_20232167_TableS3.docx]

Table S3. List of antibodies

| **Antibody** | **Company and code** | **Host** | **Dilution** |
| --- | --- | --- | --- |
| Anti-Beta Actin | Sigma-Aldrich, A2228 | mouse | 1/10000 (WB) |
| Anti-Histone H3 | Cell Signaling, 9715 | rabbit | 1/3000 (WB) |
| Anti STING | Cell Signaling, 13647 | rabbit | 1/1000 (WB) |
| Anti P-STAT1 | Cell Signaling, 7649S | rabbit | 1/500 (WB) |
| Anti-MLKL (phospho S358) | Abcam, ab187091 | rabbit | 1/500 (WB) |
| Anti-USP18 | Cell Signaling Technology, 4813T | rabbit | 1/750 (WB) |
| Anti STING | Abcam, ab181125 | rabbit | 1/1000 (IF) |
| Anti GM130, clone: 35/GM130 | BD Biosciences, 610823 | mouse | 1/100 (IF) |
| Anti Cleaved Caspase 3 (Asp175) | Cell Signaling, 9661S (BK9661S) | rabbit | 1/200 (IF) |
| Anti phospho-Histone H2A.X (Ser139), clone JBW301 | Merck, 05-636 | mouse | 1/200 (IF) |
| Alexa Fluor 488 anti-mouse IgG | Thermo Scientific A-21202 | donkey | 1/1000 (IF) |
| Alexa Fluor 488 anti-rabbit IgG | Thermo Scientific A-21206 | donkey | 1/500 (IF) |
| Alexa Fluor 555 anti-mouse IgG | Thermo Scientific A-31570 | donkey | 1/500 (IF) |
| Alexa Fluor 555 anti-rabbit IgG | Thermo Scientific A-31572 | donkey | 1/1000 (IF) |
