# Supplementary material for: A novel STING variant triggers endothelial toxicity and SAVI disease
Source: J Exp Med. 2024 Jul 2;221(9):e20232167. doi: 10.1084/jem.20232167 (PMC11217899; doi:10.1084/jem.20232167)
Supplement: Table S4 — lists drugs. [file JEM_20232167_TableS4.docx]

Table S4. List of drugs

| **Drug** | **Company and code** | **Working concentration** |
| --- | --- | --- |
| H151 | Invivogen, inh-h151 | 5 µM |
| 2’3’-cGAMP | Invivogen, tlrl-nacga23 | 10 ng/µl |
| Anti-IFNAR2 antibody (clone MMHAR-2) | PBL Assay Science, 21385-1 | 10 ng/µl |
| BX795 (TBK1 inhibitor) | Invivogen, tlrl-bx7 | 5 µM |
| Ruxolitinib (JAK1/2 Inhibitor) | Invivogen, tlrl-rux | 5 µM |
| Poly(I:C) HMW | Invivogen, tlrl-pic | 50 ng/µl |
| Human IFN-Alpha 2b (Alpha 2) | PBL Assay Science, 11105 | 1 Unit/µl |
| Anti-hIFN-γ-IgA | Invivogen, hifng-mab7-02 | 1 ng/µl |
| Infliximab | Merck, 170277-31-3 | 20 µg/ml |
| ZVAD | Invivogen, tlrl-vad | 30 µM |
| Bafilomycin A1 | Sigma, HY-100558 | 100 nM |
| 3-Methyladenine (3-MA) | Invivogen, inh-3ma-2 | 5 mM |
| Vixarelimab | MedChem Express, HY-P99519 | 0.1 ng/µl |
