# Supplementary figures and images for: A novel STING variant triggers endothelial toxicity and SAVI disease
Source: J Exp Med. 2024 Jul 2;221(9):e20232167. doi: 10.1084/jem.20232167 (PMC11217899; doi:10.1084/jem.20232167)

Row 1

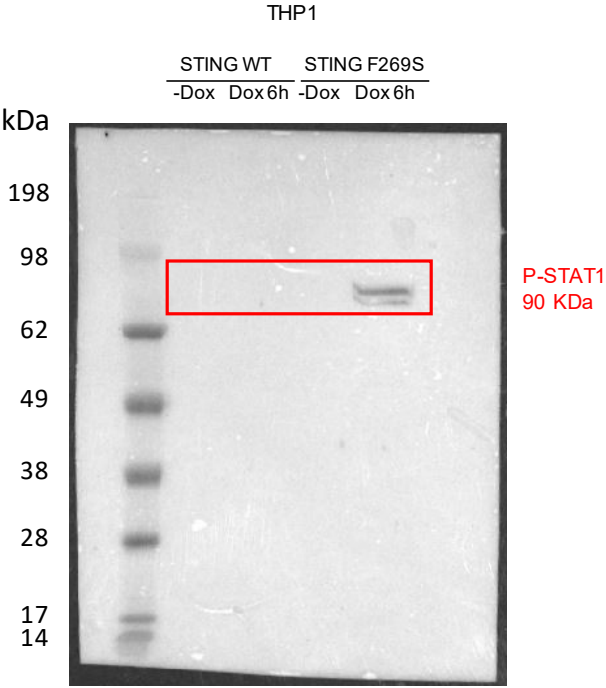

Row 2

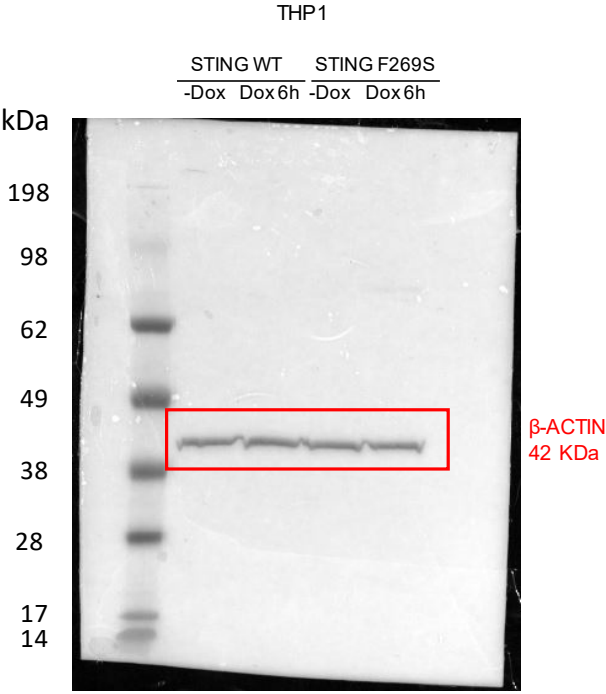

Supplement: SourceData F3 — contains original blots for Fig. 3. [file JEM_20232167_SourceDataF3.pdf]

Row 1

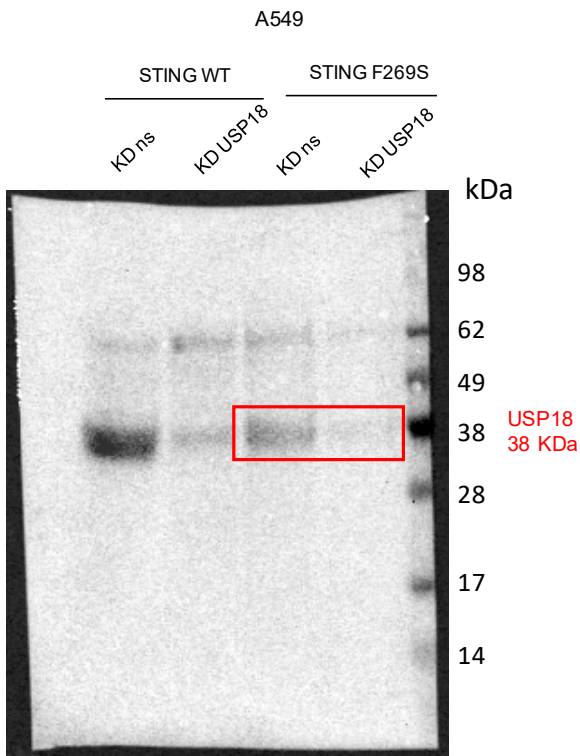

Row 2

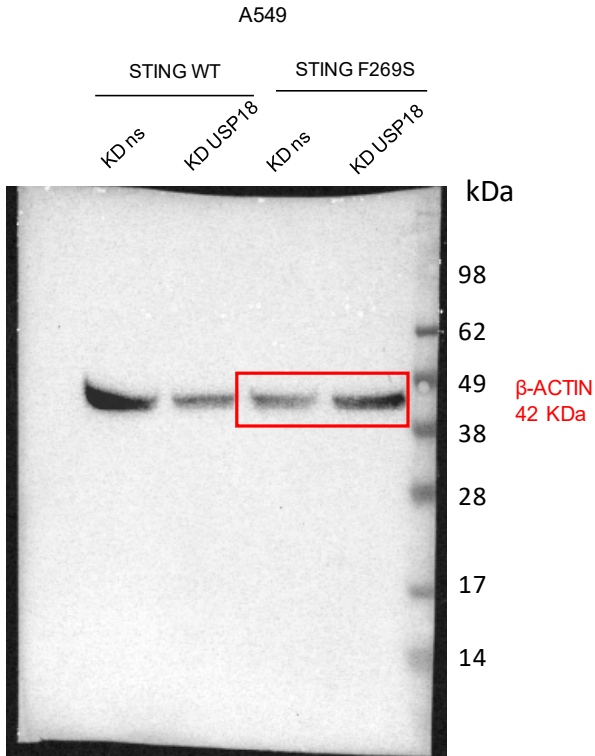

Supplement: SourceData F7 — contains original blots for Fig. 7. [file JEM_20232167_SourceDataF7.pdf]

Row 1

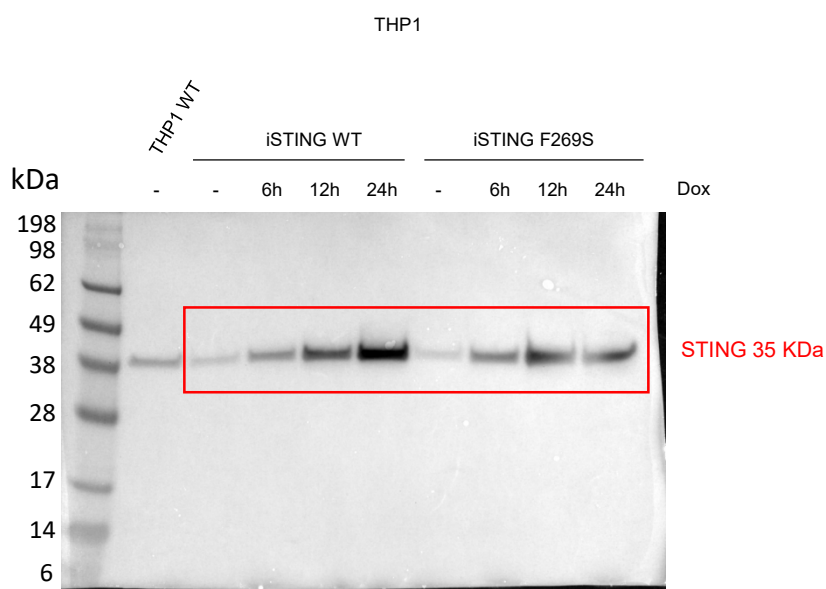

Row 2

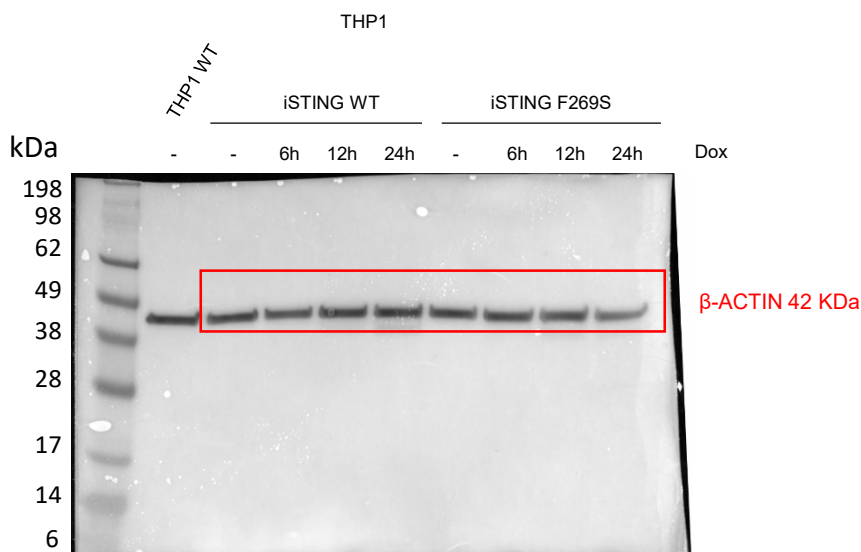

Row 1

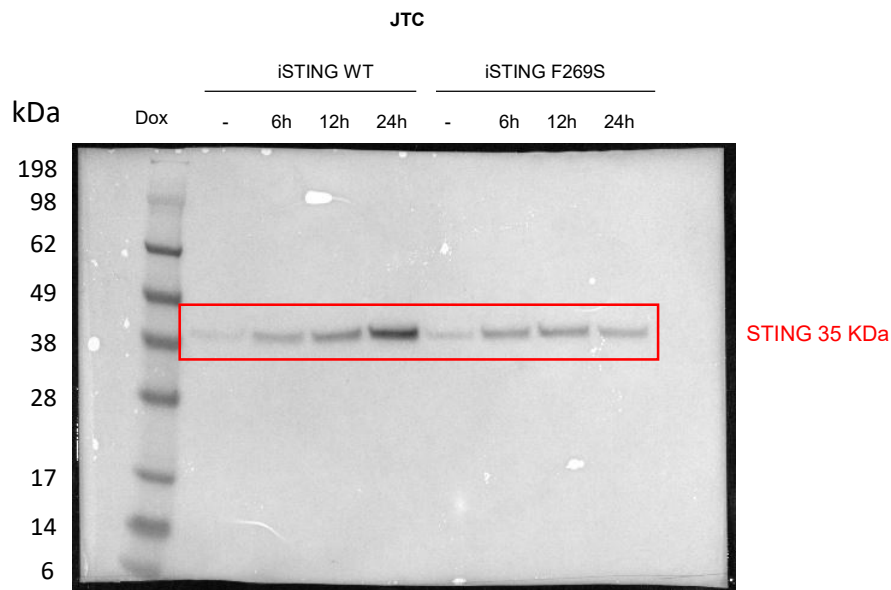

Row 2

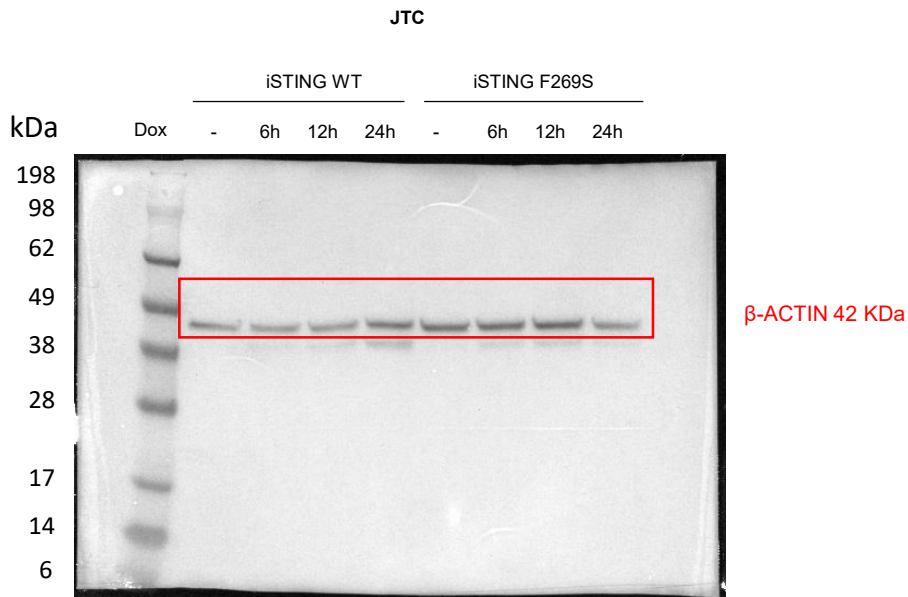

Row 1

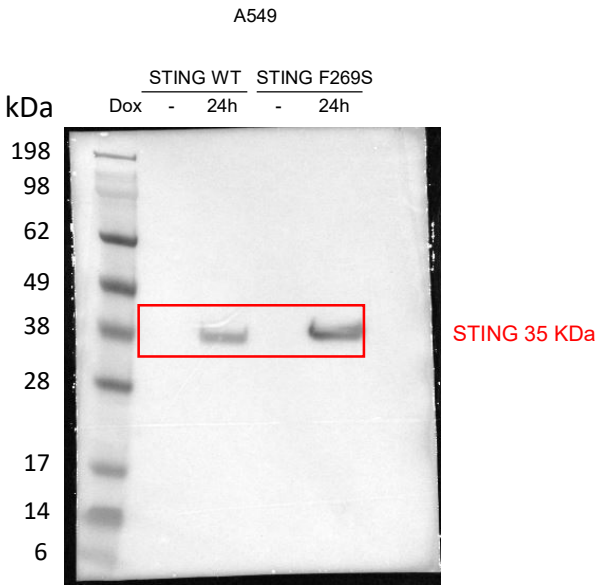

Row 2

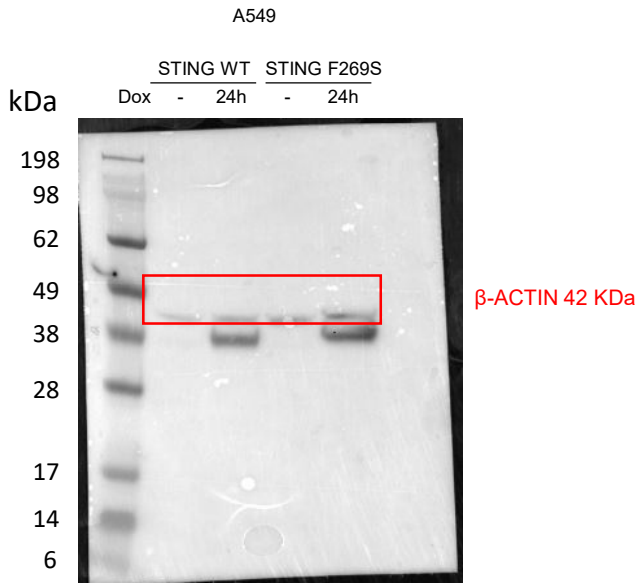

Row 1

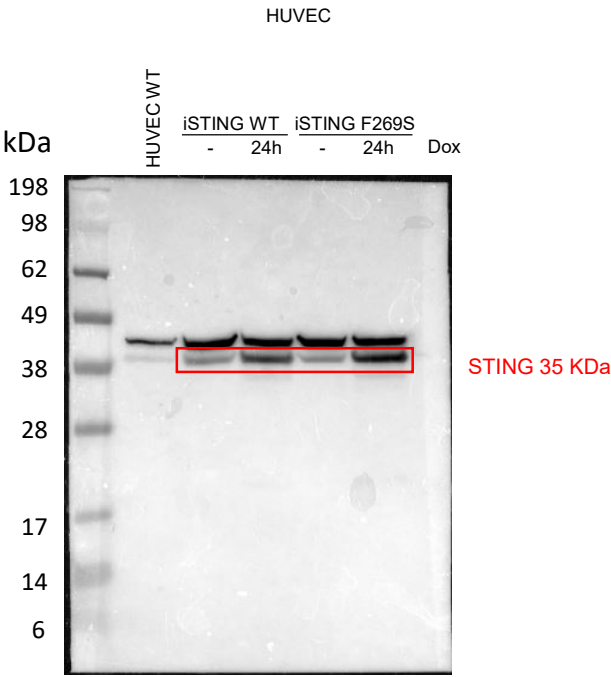

Row 2

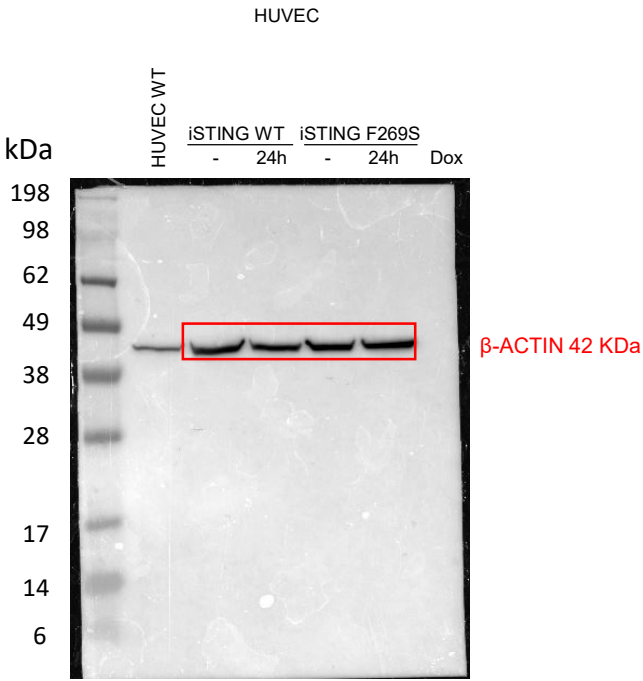

Supplement: SourceData FS2 — contains original blots for Fig. S2. [file JEM_20232167_SourceDataFS2.pdf]

Row 1

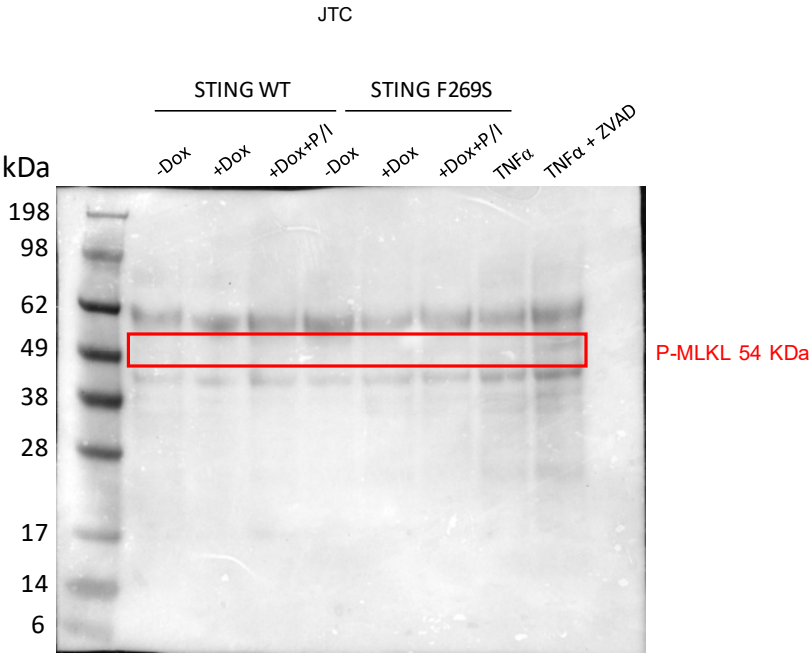

Row 2

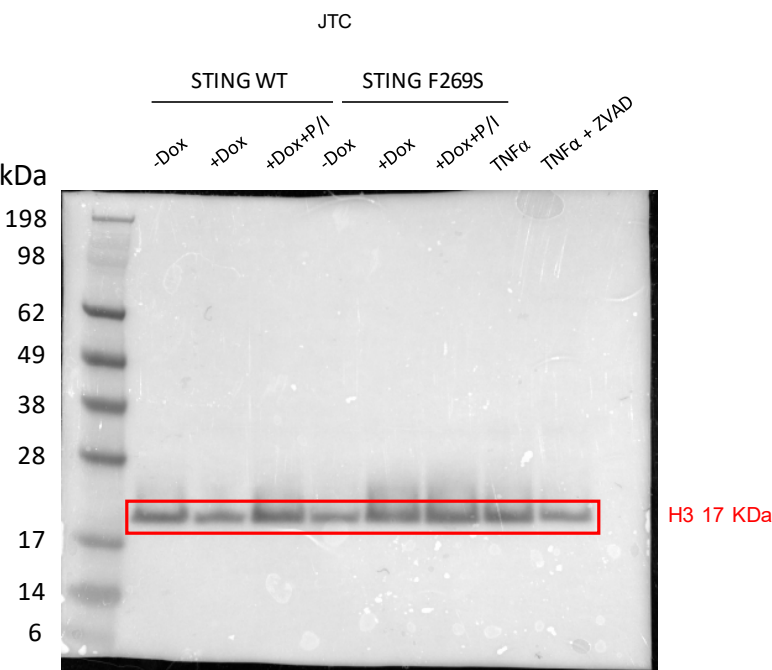

Supplement: SourceData FS3 — contains original blots for Fig. S3. [file JEM_20232167_SourceDataFS3.pdf]
